# PTEN loss drives p53 LOH and immune evasion in a novel urothelial organoid model harboring p53 missense mutations

**DOI:** 10.1038/s41388-025-03311-5

**Published:** 2025-02-22

**Authors:** Akihiro Hamada, Yuki Kita, Toru Sakatani, Kenji Nakamura, Hideaki Takada, Ryosuke Ikeuchi, Shuhei Koike, Norihiko Masuda, Kaoru Murakami, Takeshi Sano, Takayuki Goto, Ryoichi Saito, Yuki Teramoto, Masakazu Fujimoto, Narumi Hatano, Mayumi Kamada, Osamu Ogawa, Takashi Kobayashi

**Affiliations:** 1 Department of Urology, Kyoto University Graduate School of Medicine, Kyoto, Japan; 2 Department of Urology, Rakuwakai Otowa Hospital, Kyoto, Japan; 3 Department of Urology and Andrology, Kansai Medical University, Osaka, Japan; 4 Department of Diagnostic Pathology, Kyoto University Graduate School of Medicine, Kyoto, Japan; 5 Department of Biomedical Data Intelligence, Kyoto University Graduate School of Medicine, Kyoto, Japan; 6 Department of Urology, Otsu Red Cross Hospital, Shiga, Japan

**Keywords:** Bladder cancer, Disease model

## Abstract

Despite missense mutation accounts for over 60% of *p53* alterations while homozygous deletion (HOM) for only 5% or less in advanced bladder cancer cases, most of the previously reported mouse models are deficient of p53. Accordingly, few studies have addressed the mechanisms of missense mutation occurrence and its functional advantage over HOM in bladder cancer development. Organoids derived from Krt5-expressing mouse urothelium (K5-mUrorganoid) demonstrated the crucial role of *Pten* loss in driving loss of wild-type allele of *Trp53* (*Trp53*^*R172H/LOH*^), which conferred tumorigenic ability to K5-mUrorganoid in athymic mice. These tumors recapitulated the histological and genetic characteristics of the human basal-squamous subtype bladder cancer. Both *Trp53*^*R172H/Δ*^*; Pten*^*Δ/Δ*^ and *Trp53*^*Δ/Δ*^*; Pten*^*Δ/Δ*^ K5-mUrorganoids formed tumors in athymic mice, whereas only *Trp53*^*R172H/Δ*^*; Pten*^*Δ/Δ*^ K5-mUrorganoid formed tumors even when directly inoculated in immunocompetent syngeneic mice. The absence of wild-type *Trp53* was associated with upregulation of proliferative signaling, and the presence of a mutant *Trp53* allele was associated with immune-excluded microenvironment. This study highlights the functional significance of *p53* mutant LOH in bladder carcinogenesis conferring several hallmarks of cancer such as sustaining proliferative signaling and avoiding immune destruction, thus provides a novel immunocompetent mouse model of urothelial carcinoma harboring *p53* mutations as a novel tool for cancer immunology research.

## Introduction

Bladder cancer was the tenth most prevalent cancer globally in 2020, with 573,000 new cases and 213,000 deaths [1, 2]. Most bladder cancer cases are pathologically classified as urothelial carcinomas, categorized as the non-muscle-invasive bladder cancer (NMIBC) or muscle-invasive bladder cancer (MIBC) type. MIBC comprises about 25% of bladder cancer cases, with regionally invasive disease and metastatic disease patients having 50% and 15% 5-year survival rates, respectively [3]. Unfortunately, the prognosis of MIBC patients has not significantly improved over recent decades. While immune checkpoint inhibitors targeting programmed cell death 1 (PD-1)/programmed cell death-ligand 1 (PD-L1) have been approved for advanced bladder cancer, their response rate is limited to 20–30% of patients [4]. Because of this, there are urgent needs to identify factors that can define response/failure and to establish novel combination therapies that can enhance the efficacy of PD-1/PD-L1 antibodies. However, an incomplete understanding of bladder carcinogenesis and its associated immune evasion mechanisms is the greatest barrier for these efforts [5]. Therefore, reliable syngeneic models for researching bladder cancer tumorigenesis mechanisms and the tumor immune microenvironment are required [6].

In bladder cancer, *p53* mutations are the most prevalent, with over 60% being missense mutations [7]. Despite this, no de novo carcinogenesis bladder cancer mouse models involving a *p53* missense mutation have been reported, whereas *p53* knockout models have been documented [8, 9]. In addition to p53 and the cell cycle, RTK-RAS-PI3K pathway and epigenetics-related gene mutations are relevant in human MIBC [7]. PTEN copy number loss is common in MIBC and is linked to reduced RNA expression levels [10]. Indeed, several reports have shown that dual knockout of *Trp53* and *Pten* conferred tumorigenic ability to mouse bladder cells [8, 9, 11]. However, reports on the causal relationship between mutations in epigenetic-related genes, such as *KMT2C*, and urothelial carcinogenesis are extremely limited [12] compared with reports on mutation frequencies [7, 13]. Hence, we became interested in whether the additional loss of *Pten* and/or *Kmt2c* in the context of a *Trp53* missense mutation could support bladder carcinogenesis.

Previous studies from our group and others have demonstrated that cytokeratin 5 (Krt5)-expressing urothelial cells can be the originating cell type of basal subtype MIBC [14, 15]. However, because Krt5-expressing cells exist throughout the body, it has been a challenge to induce mutations specifically in the urothelium. There have been only a few reports showing that intravesical injection of Adeno Krt5-Cre in *flox* mice or 4′-hydroxy-tamoxifen (4′-OHT) treatment of Krt5-CreERT2-expressing *flox* mice could enable Krt5+ cell-specific gene recombination and tumor growth [9]. One obstacle for intravesical vector injection is the barrier of the glycosaminoglycan layer, which prevents vector access to the Krt5-expressing basal layer [16]. Another problem is that systemic treatment with tamoxifen can induce mutations in Krt5-expressing cells throughout the whole body that results in premature mouse death because of tumor development. Krt5-expressing cells in multiple tissues have a period required for carcinogenesis that is shorter than that of the bladder [11].

In the present study, we addressed these hurdles by using an organoid culture system. Inducing in vivo gene recombination in donor mice enabled selective collection of Krt5-expressing cells of urothelial lineage for organoid culture. Gene editing using the CRISPR/Cas9 system is quite straightforward in organoids. After returning the gene-edited cells into the mice and evaluating their tumorigenicity, we could verify the contribution of these specific genes to carcinogenesis. Using this system, we demonstrate that cells of the Krt5-expressing urothelial lineage can recapitulate tumors with features of the human MIBC basal-squamous subtype via organoids. Additionally, the present study shows that loss of heterozygosity (LOH) of *Trp53* is essential in the context of *Trp53* missense mutations.

## Results

### Establishment of *Trp53* mutant K5-mUrorganoids as a model of human bladder cancer that undergoes autonomous enrichment toward *Trp53* mutant LOH during tumorigenesis

To investigate the common driver genes among human and murine MIBC, we performed WES using tumor tissues obtained from human MIBC (*n* = 10) and murine bladder cancer induced by N-butyl-N-(4-hydroxybutyl)-nitrosamine (BBN) (*n* = 6). In addition to recurrent p53 mutations (50% in human MIBC and 67% in mouse MIBC), we observed frequent mutations in genes involved in ASC-2/NCOA6 complex (ASCOM) [12] (Fig. S1). Among those, a *KMT2C* (*Kmt2c*) alteration was most common with 40% (4/10) of human MIBC tumors and 83% (5/6) of BBN-induced murine bladder cancer samples (Fig. S1), which were consistent with those in a previous report from TCGA [13]. Using these findings together with insights from a previously reported bladder cancer mouse model [8], we initially selected *Kmt2c* and *Pten* as additional target genes to generate a *p53* missense mutation-based mouse model. Furthermore, we hypothesized that Krt5+ urothelial cells could be the cell of origin of basal subtype MIBC according to previous reports from our group and others [14, 15].

**Fig. 1:**
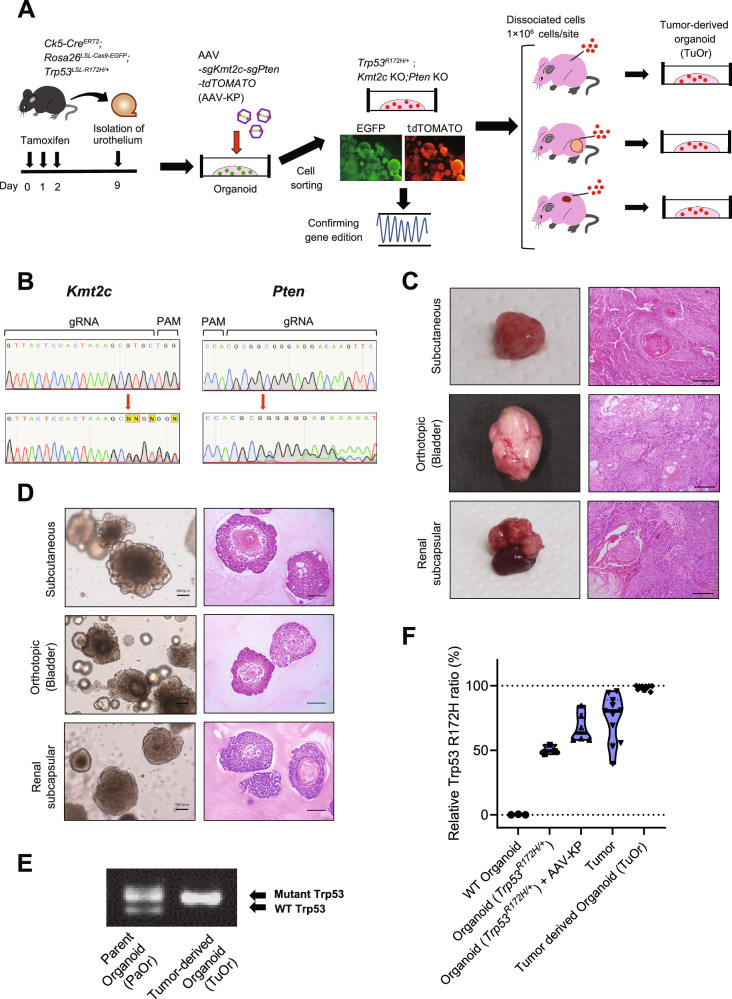
Establishment of *Trp53*-mutant K5-mUrorganoids as a model of human bladder cancer that undergoes autonomous enrichment toward *Trp53-mutant* LOH during tumorigenesis. **A** Schematics of establishing K5-mUrorganoids. Genetically engineered mice with the indicated genotype were treated with tamoxifen orally for 3 consecutive days, and then the bladder urothelial cells were collected and subjected to 3D organoid culture (K5-mUrorganoid). CRISPR/Cas9-based gene editing was performed using an adeno-associated virus (AAV) expressing single-guide (sg)RNA. K5-mUrorganoids were then subjected to ex vivo evaluation or to subcutaneous, orthotopic, and renal subcapsular inoculation for in vivo tumorigenicity assays. **B** Representative images of Sanger sequencing showing successful gene editing at the targeted positions (red arrows) by guide RNAs (gRNAs) for Kmt2c (left) and Pten (right). PAM, proto-spacer adjacent motif. **C** Representative macroscopic (left) and microscopic (right, hematoxylin and eosin (H&E) staining) images of tumors derived from *Trp53*^*R172H/+*^*; Kmt2c*-*KO; Pten KO* K5-mUrorganoids inoculated in athymic mice at the indicated sites. Note that the tumors exhibit histological characteristics of human muscle-invasive bladder cancer (MIBC) with squamous differentiation. Scale bars indicate 100 μm. **D** Representative bright-field (left) and H&E (right) microscopic images of tumor-derived organoids (TuOrs). The TuOrs retained the histological phenotype, including keratin pearl structures. Scale bars indicate 100 μm. **E** Representative gel image of genomic PCR for *Trp53* status in parent *Trp53*^*R172H/+*^*; Kmt2c-KO; Pten KO* K5-mUrorganoids (PaOr, left) and TuOrs (right). Note that the band for wild-type (WT) *Trp53* was undetectable in TuOrs, whereas the band for mutant *Trp53* was thickened. **F** Digital PCR results of WT organoids, organoids with *Trp53*^*R172H/+*^, organoids with *Trp53*^*R172H/+*^*; Kmt2c KO; Pten KO*, tumors, and TuOrs. See also Supplementary Figs. S1–S5.

To establish a bladder cancer mouse model from these hypotheses, we used organoids derived from genetically engineered mice (GEM) (Fig. 1A). First, *Krt5*^*CreERT2/+*^*; Rosa26*^*LSL-Cas9-EGFP*^*; Trp53*^*LSL-R172H/+*^ mice were treated with tamoxifen for 3 days for Krt5-expressing cells, including basal and intermediate cells, in the bladder urothelium to express Cas9, R172H mutant p53, and EGFP. Seven days after tamoxifen treatment, we collected the urothelium and generated organoids (*Trp53*^*R172H/+*^ K5-mUrorganoids). The K5-mUrorganoid cells were sorted using GFP expression to aggregate only Krt5-lineage urothelial cells. These K5-mUrorganoids maintained expression of basal markers, such as Krt5 and p63 (Fig. S2). Next, cells were infected with AAV concomitantly expressing sgKmt2c and sgPten to induce gene editing using the CRISPR/Cas9 system. Sanger sequencing was used to confirm gene editing of the target regions (Fig. 1B). *Trp53*^*R172H/+*^; *Kmt2c*-*KO; Pten KO* K5-mUrorganoid cells led to tumor development in athymic mice (BALB/cAJcl-nu/nu) by subcutaneous, orthotopic, and renal subcapsular injections. The tumors demonstrated a similar histology to those of human MIBC tumors with squamous differentiation (Fig. 1C). We then generated organoids from these tumors (tumor-derived organoid; TuOr). TuOrs retained the histological characteristics of the tumors, including keratin pearl structures inside (Fig. 1D).

Genotyping PCR revealed that TuOrs lost the WT *Trp53* allele, but maintained the mutant allele, indicating LOH of *Trp53* (*Trp53*^*R172H/LOH*^, Fig. 1E). Quantitative digital PCR showed that the *Trp53* mutant allele ratio gradually increased during the tumorigenesis process and LOH cells were enriched in TuOrs (Figs. 1F and S3). Copy number analysis using WES suggested that these TuOrs had copy number loss LOH (copy number ratio: −0.5, Fig. S4A), whereas the others had a mixture of copy number neutral LOH [17] and copy number loss LOH (copy number ratio: −0.2 to −0.3, Fig. S4B). This suggested that loss of the WT *Trp53* allele favors tumorigenesis for *Trp53*-mutated K5-mUrorganoid cells.

Next, to examine the functional significance of *Kmt2c* loss and *Pten* loss in the tumorigenicity of *Trp53*^*R172H*/*Δ*^ (generated from *Trp53*^*LSL-R172H*/*flox*^ mouse by Cre recombination) K5-mUrorganoids, we designed two distinct sgRNAs for *Kmt2c* and *Pten*. The genes were edited individually using an AAV expressing one of the sgRNAs or in combination with the two sgRNAs for *Kmt2c* and *Pten*. We also prepared *Trp53*^*R172H*/*LOH*^ K5-mUrorganoids, which were enriched by treating *Trp53*^*R172H/+*^ K5-mUrorganoids with 5 μM nutlin-3, an inhibitor of murine double minute 2 (MDM2), which is the main upstream regulator of p53. After deletion of *Kmt2c* and/or *Pten*, the in vivo tumorigenicity of *Trp53*^*+/+*^, *Trp53*^*R172H*/*+*^, and *Trp53*^*R172H*/*LOH*^ K5-mUrorganoids was assessed by subcutaneous inoculation in athymic mice (*n* = 3 or 4 for each genotype, Fig. S5A). *Trp53* WT K5-mUrorganoids did not form any tumors, irrespective of the *Kmt2c* or *Pten* genotype (Fig. S5B/C). However, *Trp53*^*R172H*/*+*^ (9/16) and *Trp53*^*R172H*/*LOH*^ (11/12) K5-mUrorganoids formed tumors only when *Pten* was deleted, but irrespective of *Kmt2c* deletion (Figs. S5B/C). *Trp53* LOH changes (loss of the WT allele) were observed in 77.8% (7/9) of tumors derived from *Trp53*^*R172H*/*+*^ K5-mUrorganoids (Fig. S5D). On the contrary, *Pten*^*+/+*^ K5-mUrorganoids did not form any tumors, irrespective of the *Trp53* or *Kmt2c* genotype (Fig. S5B). These results indicate that LOH of *Trp53* and loss of *Pten* were strongly related to in vivo tumorigenesis in the context of *Trp53*-mutant K5-mUrorganoids, whereas loss of *Kmt2c* was not.

### Single nucleotide alteration with loss of the WT allele is the most frequently observed pattern in *p53*-altered human MIBC

**Fig. 2:**
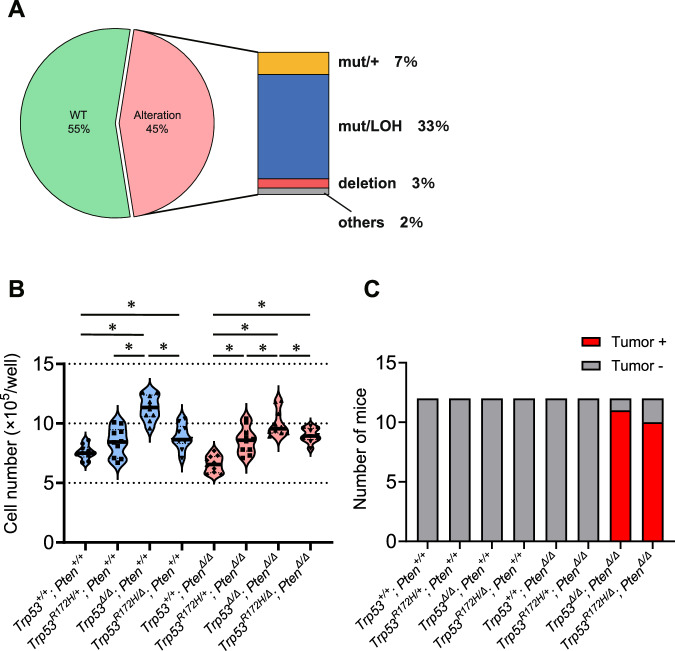
*p53* loss of heterozygosity (LOH) is common in human muscle-invasive bladder cancer (MIBC) and is required for mouse tumorigenesis in the context of *p53*-altered bladder cancer. **A** The proportion of patients according to the p53 gene status in The Cancer Genome Atlas (TCGA) data. **B** Ex vivo cell proliferation rate in organoids with distinct genotypes (*n* = 10). *P < 0.05. **C** In vivo tumor-forming rates in athymic mice for organoids with distinct genotypes (*n* = 12). See also Supplementary Figs. S6–S10.

We then investigated the *p53* status of human MIBC samples using TCGA data (Fig. S6A). *TP53* was most frequently altered gene, being reported in 45% of the MIBC patients included. Of those, 74% (33% of overall cases) harbored single nucleotide alteration with LOH (Fig. 2A). We did not find any correlation between *TP53* status and overall survival in the TCGA cohort (Fig. S6B), or with subtype, disease stage, or pathological T stage (Fig. S6C). Therefore, in cases with *TP53* alteration, a missense mutation with LOH is the most common, suggesting that there is clinical relevance for examining the functional significance of *p53* mutations with LOH in MIBC development.

### Loss of the WT *Trp53* allele and *Pten* loss confer in vivo tumorigenic ability to K5-mUrorganoids in athymic mice

To further interrogate the biological influence of the status of *Trp53* and *Pten* on K5-mUrorganoid tumorigenicity, we generated eight distinct genotypes of K5-mUrorganoids by crossing *Pten flox* and *Trp53 flox* mice: *Trp53* with WT (*Trp53*^*+/+*^), heterozygous mutant (*Trp53*^*R172H/+*^), homozygous deletion (*Trp53*^*flox/flox*^ before recombination, *Trp53*^*Δ/Δ*^ after recombination), and mutant LOH (*Trp53*^*R172H/flox*^ before recombination, *Trp53*^*R172H/Δ*^ after recombination) and *Pten* with WT (*Pten*^*+/+*^) and homozygous deletion (*Pten*^*flox/flox*^ before recombination, *Pten*^*Δ/Δ*^ after recombination) (Fig. S7A–D). Then, we evaluated the ex vivo and in vivo phenotypes, as well as the gene expression and molecular pathway profiles using bulk RNA-seq.

*Trp53*^*Δ/Δ*^ K5-mUrorganoids showed significantly higher ex vivo proliferation than any other genotype, whereas no significant differences were observed between *Trp53*^*R172H/+*^ and *Trp53*^*R172H/Δ*^ (Fig. 2B). *Pten* status did not appear to impact the K5-mUrorganoid proliferation ability. For in vivo tumorigenicity, *Trp53*^*R172H/Δ*^*; Pten*^*Δ/Δ*^ (10/12) showed an equivalent subcutaneous tumor development rate as *Trp53*^*Δ/Δ*^*; Pten*^*Δ/Δ*^ (11/12) in athymic mice, whereas the other K5-mUrorganoid genotypes, including *Trp53*^*R172H/Δ*^*; Pten*^*+/+*^ and *Trp53*^*R172H/+*^*; Pten*^*Δ/Δ*^, did not form any tumors (*n* = 12 for each genotype) (Fig. 2C). These results again indicate that loss of the WT *Trp53* allele together with *Pten* loss is required for in vivo tumor formation in athymic mice. *Trp53*^*R172H/Δ*^*; Pten*^*Δ/Δ*^ K5-mUrorganoids had a similar tumorigenic potential as *Trp53*^*Δ/Δ*^*; Pten*^*Δ/Δ*^, despite a lower proliferation ability ex vivo.

To investigate the dynamics of LOH of *Trp53* in the context of missense mutations, as well as the potential effect of *Pten* loss, we monitored the *Trp53* status over the passages using digital PCR of *Trp53*^*R172H/+*^*; Pten*^*+/+*^ and *Trp53*^*R172H/+*^*; Pten*^*Δ/Δ*^ K5-mUrorganoids. The relative ratio of the *Trp53*^*R172H*^ allele did not change significantly over passage (from P8 to P13) in *Trp53*^*R172H/+*^*; Pten*^*+/+*^ K5-mUrorganoids (Fig. S8A), while it became dominant over passage (from P4 to P9) in *Trp53*^*R172H/+*^*; Pten*^*Δ/Δ*^ K5-mUrorganoids (Fig. S8B). *Trp53*^*R172H/+*^*; Pten*^*+/+*^ K5-mUrorganoids (P11) did not form tumors in vivo, at least up to 8 weeks after inoculation (Fig. S8C, left), nor did *Trp53*^*R172H/+*^*; Pten*^*Δ/Δ*^ K5-mUrorganoids (P5) (Fig. S8C, middle). After the eighth passage, however, *Trp53*^*R172H/+*^*; Pten*^*Δ/Δ*^ K5-mUrorganoids acquired in vivo tumorigenicity (Fig. S8C, right).

The experimental results thus far indicate that, as ex vivo passage is repeated, loss of *Pten* can drive the LOH of the missense mutation allele of *Trp53*. A proliferation advantage and an accumulation of changes from passage stress do not seem to be major driving forces for LOH. For in vivo tumorigenesis, both loss of the WT allele of *Trp53* and loss of *Pten* are required, suggesting that the contribution of *Pten* loss to in vivo tumorigenesis goes beyond promoting LOH of *Trp53*.

Because LOH was induced by P9 in *Trp53*^*R172H/+*^*; Pten*^*Δ/Δ*^ K5-mUrorganoids, but not in *Trp53*^*R172H/+*^*; Pten*^*+/+*^ K5-mUrorganoids even up to P13, the difference in LOH induction between *Trp53*^*R172H/+*^*; Pten*^*Δ/Δ*^ and *Trp53*^*R172H/+*^*; Pten*^*+/+*^ does not appear to be from changes accumulated with repeated passages. On the other hand, because the *Trp53*^*R172H/+*^*; Pten*^*Δ/Δ*^ K5-mUrorganoids enriched *Trp53* mutant LOH from P4 to P9, we considered that the accumulated changes through repeated passages, in addition to the loss of *Pten*, has not been fully eliminated as a contributor to the LOH changes in *Trp53*^*R172H/+*^ K5-mUrorganoids. In other words, whether there was a contribution from multiple passages besides *Pten* loss or not remained unclear at that moment. We hypothesized that *Pten* loss is able to induce *Trp53* mutant LOH independent of multiple passages. Since cells expressing the wild-type p53 cannot survive in the presence of nutlin-3, an MDM2 inhibitor [18, 19], this means that cells proliferating under these conditions have lost the *Trp53* wild-type allele (in the case of *Trp53*^*mut/+*^, *Trp53* mutant LOH). The eight K5-mUrorganoid genotypes were cultured in the presence or absence of nutlin-3 without repeated passages. As anticipated, *Trp53*^*+/+*^*; Pten*^*+/+*^, *Trp53*^*+/+*^*; Pten*^*∆/∆*^, and *Trp53*^*R172H/+*^*; Pten*^*+/+*^ K5-Urorganoids scarcely formed organoids in the presence of nutlin-3. However, *Trp53*^*R172H/+*^*; Pten*^*∆/∆*^ K5-Urorganoids were able to grow in the presence of nutlin-3 at an approximately 50% rate compared with that in the absence of nutlin-3 (Fig. S9A/B).

K5-Urorganoids with the original *Trp53*^*R172H/+*^*; Pten*^*∆/∆*^ genotype that had grown in the presence of nutlin-3 showed *p53* LOH (*Trp53*^*R172H/LOH*^, Fig. S9C) and acquired in vivo tumorigenic ability in athymic mouse (Fig. S9D). This was consistent with the *Trp53*^*R172H/LOH*^*; Pten*^*∆/∆*^ K5-Urorganoids generated from repeated passages. TuOrs were generated from each tumor developed from the *Trp53*^*R172H/+*^*; Pten*^*Δ/Δ*^ K5-mUrorganoids with nutlin-3 selection. These TuOrs showed copy number neutral LOH of *Trp53* by WES analysis (Fig. S9E). These results collectively indicate that *Pten* loss can promote LOH of *Trp53* in the context of *Trp53*^*R172H/+*^.

We previously constructed four gene sets showing distinct enrichment patterns regarding human MIBC molecular subtypes using TCGA 2017 [13]. We also demonstrated that the gene expression profile of BBN-induced bladder cancer originating from Krt5-expressing mouse urothelial cells has the highest similarity to that of the human MIBC basal-squamous subtype [15]. Specifically, GSEA showed a characteristic enrichment pattern for the basal-squamous subtype, in which gene sets 3 and 4 were highly enriched, but gene sets 1 and 2 were not [15]. We performed GSEA using these four gene sets on tumors with each genotype and compared the results with those of normal mouse urothelium. Tumors from *Trp53*^*∆/∆*^*; Pten*^*∆/∆*^, *Trp53*^*R172H/∆*^*; Pten*^*∆/∆*^, and *Trp53*^*R172H/LOH*^*; Pten*^*∆/∆*^ (from *Trp53*^*R172H/+*^*; Pten*^*∆/∆*^ with nutlin-3) K5-mUrorganoids were highly enriched in gene sets 3 (normalized enrichment score [NES]: 1.952–2.879) and 4 (NES: 2.647–2.811). These characteristics are specific to the basal-squamous subtype in TCGA 2017 (Fig. S10A/B). Tumors from *Trp53*^*R172H/LOH*^*; Kmt2c-KO; Pten KO* K5-mUrorganoids showed a relatively lower NES (1.185 for gene set 3 and 2.531 for gene set 4), which presumably reflects the cellular heterogeneity caused by the relatively random gene editing associated with the CRISPR/Cas9 system. Our results using GSEA are generally consistent with the histological findings of these tumors, which recapitulate human MIBC with squamous differentiation.

Collectively, our mouse bladder cancer model characterized by *Trp53* LOH recapitulated both the histological and genetic characteristics of the human basal-squamous subtype, which has been shown to have the most unfavorable prognosis among MIBC patients [20]. This indicates that this model is a clinically relevant and experimentally useful tool for research aiming for a better understanding of the bladder carcinogenesis process to ultimately address clinically unmet needs.

### Alterations of *Trp53* and *Pten* contribute to bladder cancer tumorigenesis through distinct mechanisms

**Fig. 3:**
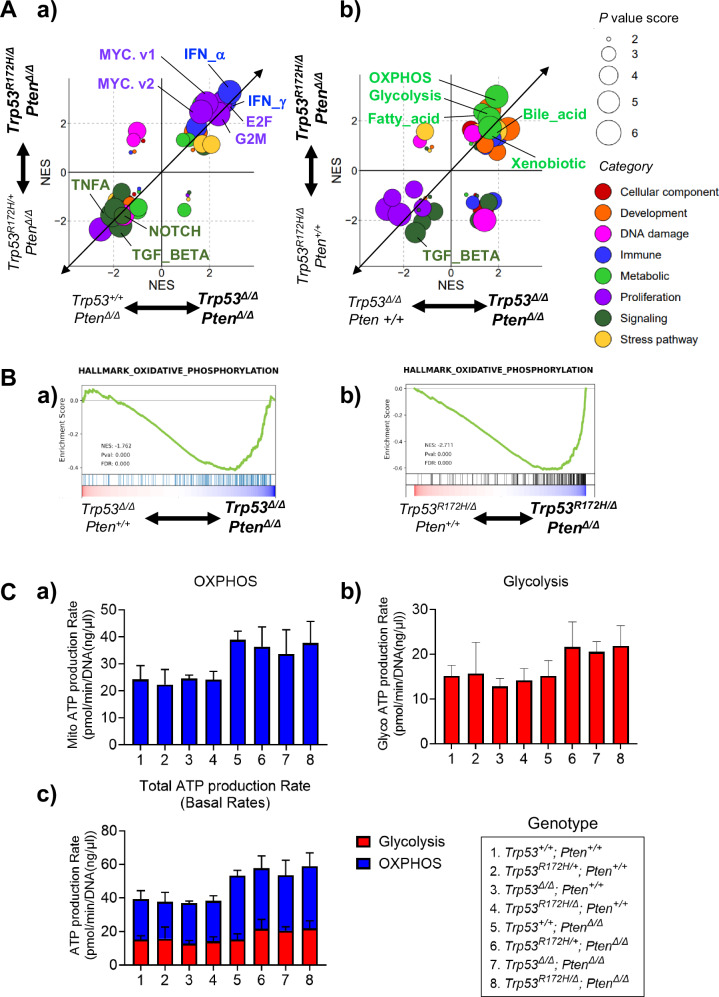
RNA-seq demonstrated distinct enrichment patterns of gene sets by loss of *Pten* and wild-type (WT) *Trp53.* **A** The enrichment patterns found using Gene Set Enrichment Analysis (GSEA) are depicted for the K5-mUrorganoid genotypes. The normalized enrichment score (NES) for each comparison between the indicated genotypes was expanded and exhibited in the scatter plots. The size of each dot indicates the sum of the *P*-value score based on the *P*-value (1, not significant; 2, *P* < 0.05; 3, *P* < 0.01) of the horizontal axis and the vertical axis. The categories were determined according to the original report [64] and differentially colored as indicated. a) Gene sets differentially enriched for the *WT Trp53* allele status. b) Gene sets differentially enriched for the *Pten* status. **B** Oxidative phosphorylation (OXPHOS) was significantly upregulated in K5-mUrorganoids with *Pten* loss. **C** Column charts showing the results of Seahorse adenosine triphosphate (ATP) assays for (a) OXPHOS, (b) glycolysis, and (c) total ATP production rates for K5-mUrorganoids with the indicated genotypes. The ATP production rate from OXPHOS and total ATP production rate were significantly higher in the organoids with *Pten* loss than in the *Pten* WT organoids (*P* = 0.002 and 0.001, respectively). See also Supplementary Figs. S11 and S12.

Next, we investigated the biological mechanisms by which the loss of the WT *Trp53* allele and deletion of *Pten* are each involved in K5-mUrorganoid tumorigenesis using RNA-seq and GSEA. K5-mUrorganoids of six different genotypes (*Trp53*^*R172H/+*^*; Pten*^*∆/∆*^, *Trp53*^*R172H/∆*^*; Pten*^*∆/∆*^, *Trp53*^*+/+*^*; Pten*^*∆/∆*^, *Trp53*^*∆/∆*^*; Pten*^*∆/∆*^, *Trp53*^*R172H/∆*^*; Pten*^*+/+*^, and *Trp53*^*∆/∆*^*; Pten*^*+/+*^) were subjected to RNA-seq analysis (*n* = 4 for each genotype). To investigate the functional loss of the WT *Trp53* allele, comparisons were made in GSEA between *Trp53*^*R172H/+*^*; Pten*^*∆/∆*^ and *Trp53*^*R172H/∆*^*; Pten*^*∆/∆*^, as well as between *Trp53*^*+/+*^*; Pten*^*∆/∆*^ and *Trp53*^*∆/∆*^*; Pten*^*∆/∆*^ (Fig. 3A-a). To investigate the functional loss of *Pten*, comparisons were made between *Trp53*^*R172H/∆*^*; Pten*^*∆/∆*^ and *Trp53*^*R172H/∆*^*; Pten*^*+/+*^, as well as between *Trp53*^*∆/∆*^*; Pten*^*∆/∆*^ and *Trp53*^*∆/∆*^*; Pten*^*+/+*^ (Fig. 3A-b).

Among the hallmark gene sets for GSEA, the proliferation-related (NES; E2F_TARGETS 2.43–2.76, G2M_CHECKPOINT 2.37–2.38, MYC_TARGETS_V1 1.87–2.81, MYC_TARGETS_V2 1.64–2.47, Fig. 3A-a, S11A/B) and interferon response (NES; INTERFERON_ALPHA_RESPONSE 2.84–3.28, INTERFERON_GAMMA_RESPONSE 2.63–2.92) gene sets were significantly enriched in the K5-mUrorganoids with *Trp53* WT allele loss, which was consistent with previous reports [21–23]. On the other hand, several tumor suppressive pathways including TNFA_SIGNALING_VIA_NFKB (NES −2.05 to −1.75), TGF_BETA_SIGNALING (NES –1.71 to –2.18), and NOTCH_SIGNALING (NES −1.66 to −1.94) were significantly inversely enriched in the K5-mUrorganoids with *Trp53* WT allele loss, which was also consistent with previous reports [24–26].

The gene sets involved in the metabolic category, including oxidative phosphorylation (OXPHOS), glycolysis, fatty acid, bile acid, and xenobiotic, were significantly enriched in the K5-mUrorganoids with *Pten* loss (Figs. 3A-b, S11C/D). On the other hand, TGF_BETA_SIGNALING (NES −1.51 to −2.49) was also inversely enriched in the K5-mUrorganoids with *Pten* loss. Additionally, we found that the reactive oxygen species (ROS) pathway was significantly upregulated on Pten loss (GOBP_REACTIVE_OXYGEN_SPECIES_METABOLIC_PROCESS; *Trp53*^*R172H/∆*^*; Pten*^*∆/∆*^ vs *Trp53*^*R172H/∆*^*; Pten*^*+/+*^; NES 1.7908, *p* < 0.01, *Trp53*^*∆/∆*^*; Pten*^*∆/∆*^ vs *Trp53*^*∆/∆*^*; Pten*^*+/+*^; NES 1.9203, p < 0.01). Because both OXPHOS and glycolysis pathway genes were enriched in K5-mUrorganoids with *Pten* loss (Fig. 3B), we then examined the mitochondrial ATP production rate using organoids with eight different genotypes (indicated in Fig. 3C). Seahorse flux analysis showed significantly higher mito (OXPHOS) ATP production rates in organoids with *Pten* loss, regardless of *Trp53* status (*Pten* status; Two-group comparison of 1–4 vs. 5–8, *P* = 0.0021, *Trp53* status; Four-group comparison of 1–2, 3–4, 5–6, and 7–8, *P* = 0.527, multiple regression analysis, Fig. 3C-a). The ATP production rate via glycolysis was also higher in K5-mUrorganoids with *Pten* loss compared with those expressing Pten, although the differences were not statistically significant (*P* = 0.0616, Fig. 3C-b). The *Trp53* status did not affect ATP production rate by glycolysis (*P* = 0.6067, Fig. 3C-b). Overall, the total ATP production rate was significantly higher in K5-mUrorganoids with *Pten* loss regardless of *Trp53* status (*Pten* status; *P* = 0.0012, *Trp53* status; *P* = 0.533, multiple regression analysis, Fig. 3C-c, see also Fig. S12).

Taken together, these results suggest that *Pten* loss and WT *Trp53* allele loss in the organoids resulted in a cellular metabolic shift and acceleration of cell proliferation that supported tumorigenesis.

### *Trp53*^R172H/∆^*; Pten*^*Δ/Δ*^ K5-mUrorganoids, but not *Trp53*^*∆/*∆^*; Pten*^*Δ/Δ*^ K5-mUrorganoids, can form tumors by direct inoculation in immunocompetent mice

**Fig. 4:**
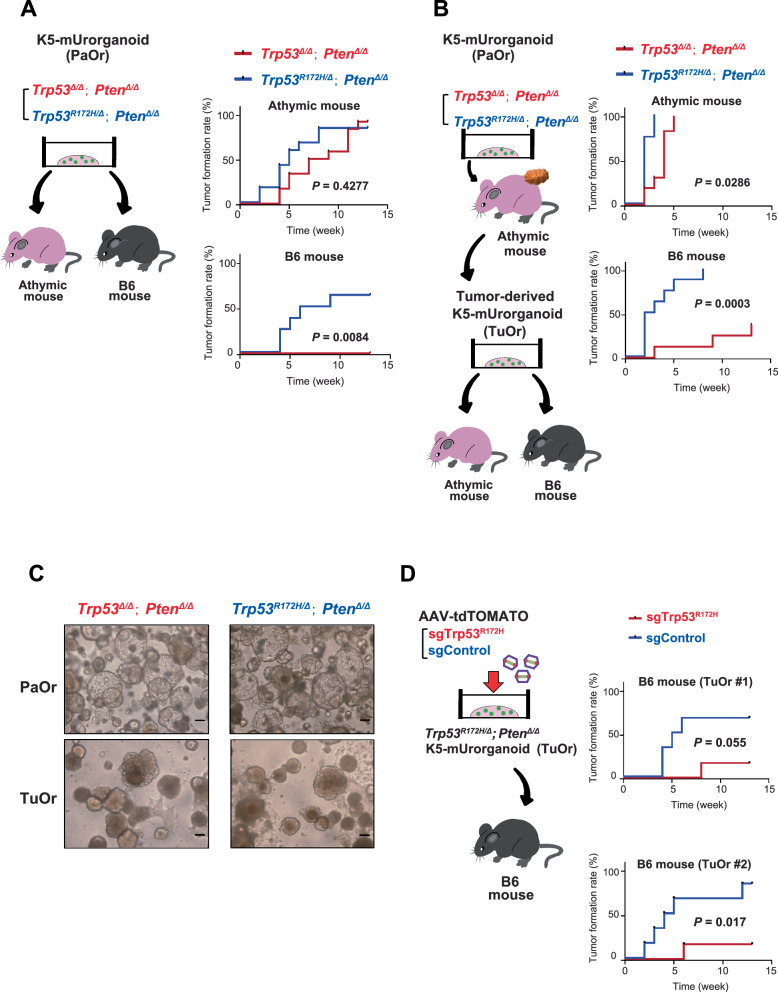
*Trp53*^*R172H/Δ*^*; Pten*^*Δ/Δ*^ K5-mUrorganoids showed higher tumor formation rates in immunocompetent mice. **A** Schematic design of the experiment (left) showing that 1 × 10^6^ cells of K5-mUrorganoids (Parent organoid; PaOr) with the indicated genotypes were inoculated subcutaneously into athymic (*n* = 12) or B6 (*n* = 8) mice. Kaplan–Meier curves (right) showing the time to tumor formation in athymic (top) and B6 (bottom) mice. **B** Schematic design of the experiment (left) showing that tumor-derived organoids (TuOrs) were generated from PaOr tumors grown in athymic mice. Here, 1 × 10^6^ cells of TuOrs with the indicated genotype were inoculated subcutaneously into athymic (n = 8) or B6 (*n* = 8) mice. Kaplan–Meier curves (right) showing the time to tumor formation in athymic (top) and B6 (bottom) mice. **C** Representative bright-field microscopic images of PaOrs and TuOrs with the indicated genotypes. Scale bars indicate 100 μm. **D** Schematic design of the experiment (left) showing that AAV-sgTrp53R172H or sgControl was used to infect two independently generated *Trp53*^*R172H/Δ*^*; Pten*^*Δ/Δ*^ TuOrs. These organoids were inoculated subcutaneously into athymic (*n* = 6) or B6 (*n* = 6) mice. Kaplan–Meier curves (right) showing the time to tumor formation for each TuOr. See also Supplementary Figs. S13 and S14.

Various immunotherapies, including PD-1/PD-L1-targeting drugs, have been introduced for treating urothelial cancers [4, 27], and thus there is an increasing need for animal models with normal immune systems. Therefore, we investigated whether the *Trp53*^*R172H/∆*^*; Pten*^*∆/∆*^ and *Trp53*^*∆/∆*^*; Pten*^*∆/∆*^ K5-mUrorganoids were able to form tumors in the immunocompetent B6 (C57BL/6NJcl) mice (*n* = 8 each, Fig. 4A, left). Both *Trp53*^*R172H/∆*^*; Pten*^*∆/∆*^ and *Trp53*^*∆/∆*^*; Pten*^*∆/∆*^ K5-mUrorganoids did not significantly differ in the tumor formation rate (83.3% vs. 91.7%, *P* = 1.000, Fisher’s exact test) or time to tumor formation (5 vs. 8 weeks, *P* = 0.4277, log-rank test) in athymic mice (Fig. 4A, right top and S13A, left top). *Trp53*^*R172H/∆*^*; Pten*^*∆/∆*^ K5-mUrorganoids formed tumors in immunocompetent B6 mice at a comparable rate to those in athymic mice (62.5%, *n* = 8), whereas none of the *Trp53*^*∆/∆*^*; Pten*^*∆/∆*^ K5-mUrorganoids (*n* = 8) formed tumors in B6 mice (tumor formation rate; *P* = 0.0256, Fisher’s exact test, time to tumor formation; 7.5 weeks vs. not reached, *P* = 0.0084, log-rank test, Fig. 4A, right bottom and S13A, right top). Our results indicate that *Trp53*^*R172H/∆*^*; Pten*^*∆/∆*^ K5-mUrorganoids can genetically and morphologically recapitulate the basal-squamous human MIBC subtype, as well as be potentially useful as an experimental tool, particularly for immuno-oncology research.

We next investigated whether cells collected from the tumors formed in athymic mice had acquired a higher tumor-forming ability. For that purpose, we collected cells from tumors that originated from K5-Urorganoids (parental organoids; PaOrs) and were grown in athymic mice, then successfully generated secondary organoids (tumor-derived organoids; TuOrs, Fig. 4B, left). Both *Trp53*^*R172H/∆*^*; Pten*^*∆/∆*^ and *Trp53*^*∆/∆*^*; Pten*^*∆/∆*^ TuOrs formed tumors in athymic mice, with a shorter time to tumor formation for *Trp53*^*R172H/∆*^*; Pten*^*∆/∆*^ TuOrs than *Trp53*^*∆/∆*^*; Pten*^*∆/∆*^ TuOrs (2 vs. 4 weeks, *P* = 0.0286, log-rank test, Fig. 4B, right top and S13A, left bottom). Relative to the corresponding PaOrs, TuOrs formed tumors at comparable rates (83.3 vs. 100%, *P* = 0.4947 for *Trp53*^*R172H/∆*^*; Pten*^*∆/∆*^ K5-mUrorganoids and 91.7 vs. 100%, *P* = 1.000 for *Trp53*^*∆/∆*^*; Pten*^*∆/∆*^ K5-mUrorganoids, Fisher’s exact test), but did so more rapidly (median time to tumor formation for PaOrs vs. TuOrs; 5 vs. 2 weeks for *Trp53*^*R172H/∆*^*; Pten*^*∆/∆*^ K5-mUrorganoids, *P* = 0.0002, and 8 vs. 4 weeks for *Trp53*^*∆/∆*^*; Pten*^*∆/∆*^ K5-mUrorganoids, *P* = 0.0002, log-rank test).

In immunocompetent B6 mice, *Trp53*^*R172H/∆*^*; Pten*^*∆/∆*^ TuOrs maintained a tumor-forming ability. The *Trp53*^*∆/∆*^*; Pten*^*∆/∆*^ TuOrs acquired a tumor-forming ability, although the tumor formation rate (100 vs. 37.5%, *P* = 0.0256, Fisher’s exact test) and median time to tumor formation (2.5 weeks vs. not reached, *P* = 0.0003, log-rank test, Fig. 4B, right bottom and S13A, right bottom) were significantly lower than those of *Trp53*^*R172H/∆*^*; Pten*^*∆/∆*^ TuOrs. Relative to the corresponding PaOrs, *Trp53*^*R172H/∆*^*; Pten*^*∆/∆*^ TuOrs formed tumors at a comparable rate (62.5 vs. 100%, *P* = 0.2), but did so more rapidly (median time to tumor formation for PaOrs vs. TuOrs; 7.5 vs. 2.5 weeks, *P* = 0.0062, log-rank test). Similarly, 37.5% of *Trp53*^*∆/∆*^*; Pten*^*∆/∆*^ TuOrs formed tumors, while the corresponding PaOrs did not (*P* = 0.2, Fisher’s exact test, *P* = 0.0628, log-rank test). These results indicate that, through the tumor formation process in athymic mice, the tumor-forming ability was enhanced in both *Trp53*^*R172H/∆*^*; Pten*^*∆/∆*^ and *Trp53*^*∆/∆*^*; Pten*^*∆/∆*^ K5-mUrorganoids. Particularly, *Trp53*^*∆/∆*^*; Pten*^*∆/∆*^ K5-mUrorganoids acquired a tumor-forming ability in immunocompetent mice only after they had gone through tumor formation in athymic mice. Morphologically, PaOrs were characterized by a round regular shape with hollows, while TuOrs appeared irregular in shape and denser (Fig. 4C).

To further validate the association between the presence of the *Trp53* R172H mutant and a higher tumor-forming ability in immunocompetent mice, we next used the CRISPR/Cas9 system to knock out *Trp53* R172H expression in two independent lines of *Trp53*^*R172H/∆*^*; Pten*^*Δ/Δ*^ TuOrs derived from different tumors that were developed in athymic mice. We then evaluated the tumor-forming ability in immunocompetent B6 mice (Fig. 4D, left). Consistent with the *Trp53*^*∆/∆*^*; Pten*^*∆/∆*^ K5-mUrorganoids, TuOrs with *Trp53* R172H knocked out showed an inferior tumor-forming ability to TuOrs treated with control sgRN*A* (*Trp53*^*R172H/∆*^*; Pten*^*∆/∆*^ TuOr #1; tumor formation rate; 66.7 vs. 16.7%, *P* = 0.2424, Fisher’s exact test, time to tumor formation; 5.5 weeks vs. not reached, *P* = 0.055, log-rank test, Fig. 4D, right top and S13B, left, *n* = 6 each; *Trp53*^*R172H/∆*^*; Pten*^*∆/∆*^ TuOr #2; tumor formation rate; 83.3 vs. 16.7%, *P* = 0.0801, Fisher’s exact test, time to tumor formation; 4.5 weeks vs. not reached, *P* = 0.0017, log-rank test, Fig. 4D, right bottom and S13B, right, *n* = 6 each).

**Fig. 5:**
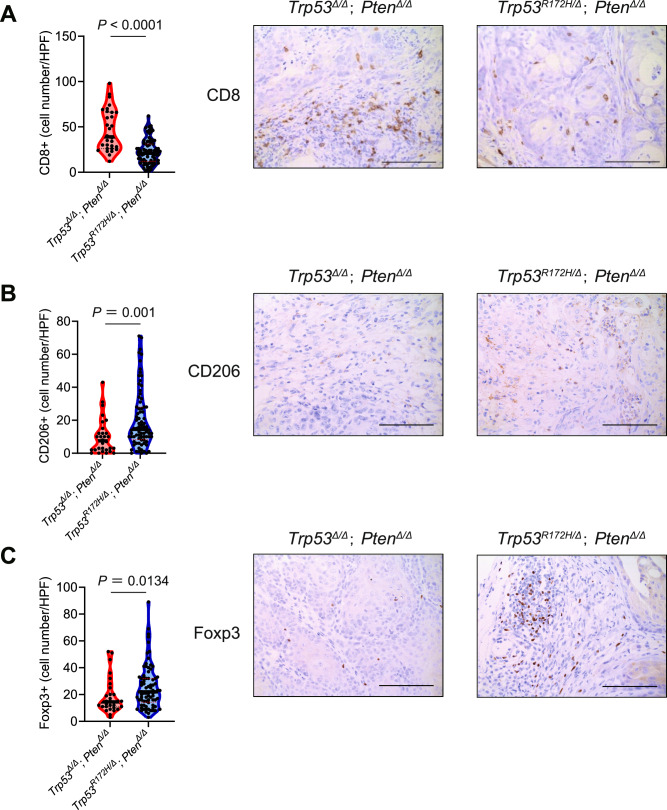
Immune microenvironment of tumors from tumor-derived organoids (TuOrs). The number of infiltrated cells (left) and representative images (right) of immunohistochemistry assays using **A** anti-CD8, **B** anti-CD206, and **C** anti-Foxp3 antibodies. Scale bars indicate 100 μm.

Next, we wanted to further explore the specific immunological mechanisms underlying the differential tumor-forming ability between *Trp53*^*R172H*^-expressing and *Trp53*-null K5-mUrorganoids, particularly in the tumor microenvironment. Using tumor tissues derived from *Trp53*^*R172H/∆*^*; Pten*^*∆/∆*^ and *Trp53*^*∆/∆*^*; Pten*^*∆/∆*^ TuOrs inoculated in B6 mice, we evaluated immune cell infiltration using IHC assays. Tumors derived from *Trp53*^*R172H/Δ*^*; Pten*^*Δ/Δ*^ TuOrs showed less infiltration of CD8+ lymphocytes (median cell number/high-power field (HPF); 39 vs. 21, *P* < 0.001, Wilcoxon test, Fig. 5A), higher M2 macrophage marker expression (CD206, 14.5 vs. 7.5, *P* = 0.001, Wilcoxon test, Fig. 5B), and higher regulatory T cell (Treg) marker expression (Foxp3, 22 vs. 14.5, *P* = 0.0134, Wilcoxon test, Fig. 5C) compared with those from *Trp53*^*Δ/Δ*^*; Pten*^*Δ/Δ*^ TuOrs. H&E staining and IHC assays for Ck5 and p63 confirmed that both *Trp53*^*R172H/∆*^*; Pten*^*∆/∆*^ and *Trp53*^*∆/∆*^*; Pten*^*∆/∆*^ TuOr-derived tumors maintained the morphological features of the human MIBC basal-squamous subtype (Fig. S14).

These results suggest that the differential tumor-forming ability between *Trp53R172H*-expressing and *Trp53*-null K5-mUrorganoids is related to immuno-oncological mechanisms. This is a potential explanation for the higher prevalence of the mutant LOH genotype compared with the homozygous deletion in *TP53* among human MIBC cases. Additionally, we have demonstrated that both *Trp53*^*R172H/Δ*^*; Pten*^*Δ/Δ*^ and *Trp53*^*Δ/Δ*^*; Pten*^*Δ/Δ*^ TuOrs can be tumorigenic in immunocompetent mice with different tumor microenvironment profiles. Taken together, the K5-mUrorganoid is a promising model of human basal-squamous MIBC that is useful for further understanding the carcinogenesis process and establishing preclinical models of immuno-oncological treatment methods for this understudied disease.

### Mutant *Trp53*-expressing organoids have decreased secretion of cytokines that mediate inflammation and CD8+ T cell activation compared with *Trp53-null* organoids

To further differentiate the immuno-oncological characteristics of *Trp53*^*R172H*^-expressing and *Trp53*-null TuOrs, we performed a cytokine array. First, we compared *Trp53*^*R172H/Δ*^*; Pten*^*Δ/Δ*^ and *Trp53*^*Δ/Δ*^*; Pten*^*Δ/Δ*^ TuOrs (comparison A) using two distinct TuOr lines for each genotype (#1; TuOr derived from female GEM, #2; TuOr derived from male GEM). Second, using two distinct TuOr lines (comparison B, #1; TuOr derived from female GEM, #2; TuOr derived from male GEM, see also Fig. 4D, left), we compared *Trp53*^*R172H/Δ*^*; Pten*^*Δ/Δ*^ K5-mUrorganoids treated with AAV-*tdTOMATO* expressing the control sgRNA (sgControl) with those treated with AAV-*tdTOMATO* expressing sgRNAs for *Trp53*^*R172H/Δ*^ (sgTrp53^R172H^).

**Fig. 6:**
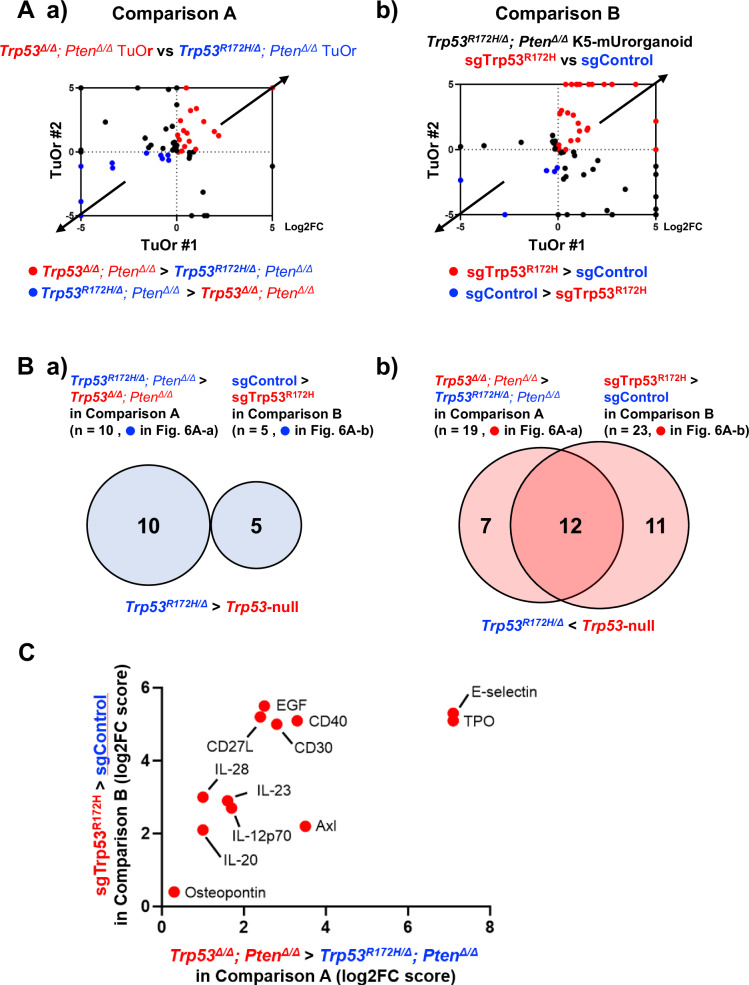
Differential cytokine profiles between *Pten*^*Δ/Δ*^ tumor-derived organoids (TuOrs) expressing mutant or no *Trp53.* **A.** a) Log2 fold-change (Log2FC) in cytokine expression levels of *Trp53*^*Δ/Δ*^; *Pten*^*Δ/Δ*^ TuOrs relative to *Trp53*^*R172H/Δ*^*; Pten*^*Δ/Δ*^ TuOrs (comparison A) from female (horizontal axis, TuOr#1) and male (vertical axis, TuOr#2) mice. b) Log2FC in cytokine expression levels of *Trp53*^*R172H/Δ*^*; Pten*^*Δ/Δ*^ TuOrs treated with sgTrp53^R172H^ relative to those treated with sgControl (comparison B). The blue dots represent the cytokines increased in TuOrs expressing *Trp53*^*R172H/Δ*^, while the red dots represent the cytokines increased in TuOrs expressing no *Trp53*. **B**. Venn diagrams for (a) upregulated and (b) downregulated cytokines in K5-mUrorganoids expressing mutant *Trp53* compared with those expressing no *Trp53* commonly between comparisons A and B. **C**. List of 12 cytokines that were downregulated in K5-mUrorganoids expressing mutant *Trp53* compared with those expressing no *Trp53* commonly between comparisons A and B. The bars represent Log2FC. See also Supplementary Figs. S15.

The comparison A helped us identify 10 cytokines that were elevated in *Trp53*^*R172H/Δ*^*; Pten*^*Δ/Δ*^ TuOrs compared with *Trp53*^*∆/Δ*^*; Pten*^*Δ/Δ*^ TuOrs (Fig. 6A-a, blue dots, and Supplementary Table S2A-a), as well as 19 cytokines that were decreased in *Trp53*^*R172H/Δ*^*; Pten*^*Δ/Δ*^ TuOrs compared with *Trp53*^*∆/Δ*^*; Pten*^*Δ/Δ*^ TuOrs (Fig. 6A-b, red dots, and Supplementary Table S2A-b). Similarly, the comparison B indicated that five cytokines were elevated in *Trp53*^*R172H/Δ*^*; Pten*^*Δ/Δ*^ K5-mUrorganoids treated with sgControl compared with those treated with sgTrp53^R172H^ (Fig. 6A-b, blue dots, and Supplementary Table S2B-a). Additionally, 23 cytokines were decreased in *Trp53*^*R172H/Δ*^*; Pten*^*Δ/Δ*^ K5-mUrorganoids treated with sgTrp53^R172H^ compared with those treated with sgControl (Fig. 6A-b, red dots, and Supplementary Table S2B-b).

No cytokine was found to be commonly elevated between *Trp53*^*R172H/Δ*^*; Pten*^*Δ/Δ*^ TuOrs (comparison A, *n* = 10) and *Trp53*^*R172H/Δ*^*; Pten*^*Δ/Δ*^ K5-mUrorganoids treated with sgControl (comparison B, *n* = 5) (Fig. 6B-a and Supplementary Table S2). In contrast, 12 cytokines were increased in both *Trp53*^*Δ/Δ*^*; Pten*^*Δ/Δ*^ TuOrs compared with *Trp53*^*R172H/Δ*^*; Pten*^*Δ/Δ*^ TuOrs (comparison A, *n* = 19) and *Trp53*^*R172H/Δ*^*; Pten*^*Δ/Δ*^ K5-mUrorganoids treated with sgTrp53^R172H^ compared with those treated with sgControl (comparison B, *n* = 23) (Fig. 6B-b, C). Among these 12 cytokines, CD27L, IL-12p70, IL-23, and Osteopontin have been reported to have functions related to inflammation or the recruitment and activation of CD8+ T cells [28–31]. Some previous reports have shown that mutant p53 variants transcriptionally regulate genes related to tumor immune microenvironment differently from wild-type p53 [32, 33]. We also performed qPCR using parent organoids with eight distinct genotypes to decipher R172H transcriptional differences from WT p53. We found that some of those genes including *Lgals9* (coding Galectin 9, a ligand of TIM3) and *Ccl5* were differentially expressed according to *Trp53* status, but regardless of *Pten* status (Fig. S15), which was also correlated with tumor-forming ability in immune-competent mice.

Collectively, the results of our multiple comparisons between mutant *Trp53*-expressing and *Trp53-null* organoids showed decreased secretion of cytokines that mediate inflammation and CD8+ T cell activation in mutant *Trp53*-expressing organoids compared with *Trp53-null* organoids. These data are consistent with a higher prevalence of *TP53*-mutant LOH in human MIBC tumors and a higher in vivo tumor-forming ability of *Trp53*-mutant LOH organoids, particularly in immunocompetent mice, compared with p53-null cases.

## Discussion

**Fig. 7:**
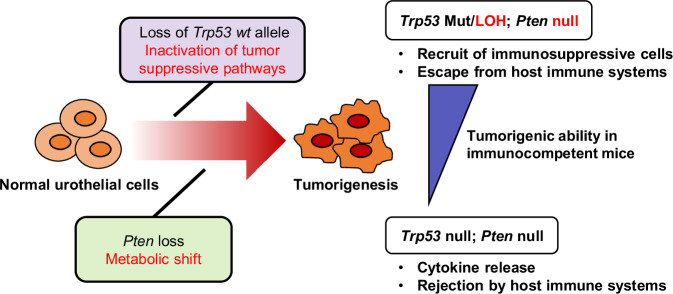
Schematic of the tumorigenic process of the K5-mUrorganoid model. *Pten* loss in normal urothelial cells causes a metabolic shift, while a *Trp53* missense mutation followed by loss of heterozygosity (LOH) promotes cell proliferation and a tumor-forming ability. The K5-mUrorganoids with mutant *Trp53* with LOH release relatively lower levels of cytokines compared with the Trp53-null organoids, creating an immune-cold tumor microenvironment that favors tumorigenesis.

In the present study, we have demonstrated the clinical and biological significance of *p53* missense mutations with LOH (loss of the *p53* WT allele), as well as functional loss of *Pten*. Furthermore, with our findings, we have proposed a novel immunocompetent animal model for human MIBC that can be easily used to test hypotheses regarding the molecular mechanisms of urothelial cancer biology, particularly in the context of immuno-oncological research. In immunocompetent mice, tumors from *Trp53* ^*R172H/Δ*^*; Pten*^*Δ/Δ*^ organoids had higher tumor formation rates with less immune cell infiltration compared with *Trp53*^*Δ/Δ*^*; Pten*^*Δ/Δ*^ organoids, suggesting that *Trp53* mutations with LOH provide an advantage to the developing tumor compared with the homozygous loss of *Trp53* (Fig. 7).

Studies have shown that there is a high prevalence of *p53* LOH in conjunction with missense mutations, as evidenced by 93% of human cancers harboring a *p53* mutation with loss of the WT *p53* allele [34, 35]. The results of the TCGA analysis in the present study showed that at least 80% of cases with a *p53* mutation demonstrated LOH. Nonetheless, to date, bladder cancer research has almost exclusively used *Trp53*-null models. Additionally, research on the difference in biological significance between *Trp53* missense LOH and *Trp53*-null is limited. This study has provided an explanation to the phenomenon in which mutant LOH is much more dominant than deletion for p53 gene in human carcinoma by combining not only the tumor intrinsic mechanisms but also the effects on the tumor immunological microenvironment.

Previous reports on other malignant diseases have shown that cells with *Trp53* LOH became increasingly predominant under stress conditions, such as passaging, irradiation, and in vivo transplantation, as a part of a selection process in tumor formation [36–39]. This suggests that the p53 LOH, in conjunction with missense mutations, universally confers an advantage to cancer cells in escaping biological stress. However, the underlying mechanisms appear to be diverse or context dependent. Most recently, Nakayama et al. demonstrated the enrichment of *Trp53* LOH cells in metastatic tumors in a colon cancer mouse model [40, 41]. Compared with *Trp53-null* cells, *Trp53* LOH cells showed upregulation of the stem cell signature, with enrichment of inflammatory and growth factor/MAPK pathways. This facilitated dormant cell survival and the tumor-initiating ability.

Our work here using K5-mUrorganoids is consistent with previous reports regarding the enrichment of *Trp53* LOH cells through passages, which can eventually confer the in vivo tumor-forming ability. According to the pathway analysis of K5-mUrorganoids, loss of WT *p53* was associated with an enrichment of genes involved in the c-Myc pathway. WT p53 can reportedly suppress c-Myc at multiple levels, including transcriptionally [22, 23] and post-transcriptionally [21]. Additionally, several gain-of-function p53 mutants, including R172H, have been reported to enhance *c-Myc* expression via activation of the Wnt/β-catenin pathway [42]. Additionally, our pathway analyses suggest that loss of *Trp53* wild-type allele induce inactivation of various tumor-suppressing pathways.

The present study has demonstrated a new example of tumor-promoting mechanisms, which have attracted attention in recent years [43–45], that cancer driver gene mutations and activation of cancer-promoting signal transduction pathways modulate not only tumor-intrinsic phenotypic changes but also host tumor immunity in the tumor microenvironment. The *Trp53*^*R172H/Δ*^*; Pten*^*Δ/Δ*^ K5-mUrorganoids demonstrated a higher engraftment rate than the *Trp53*^*Δ/Δ*^*; Pten*^*Δ/Δ*^ strain in immunocompetent syngeneic mice, whereas these two genotypes exhibited a nearly equivalent engraftment rate in athymic mice. The high frequency of *Trp53* missense LOH in human MIBC cases may be related to tumor immune escape-related mechanisms. Our further investigation revealed that the *Trp53* missense LOH tumors had lower cytokine release, less infiltration of CD8+ T cells, and higher infiltration of immunosuppressive cells, including M2 macrophages and Tregs. Our results are supported by previous reports, which showed that mutant *p53* can suppress anti-tumor immune responses [46]. Additionally, tumors with mutant *p53* were found more likely to be immune-excluded compared with *p53*-null tumors [47, 48]. Various mechanisms for this phenomenon have been proposed, including suppression of innate immune responses [47], inhibition of cytokine release [48], and promotion of immunosuppressive neutrophil recruitment [49]. The impact of *Trp53* status upon epigenetic regulation may also be intriguing based on our WES results and the previous report [12]. However, the exact mechanism may vary depending on the context [50].

Our current findings are not limited to the modeling of human MIBC with *p53* missense LOH or its facilitating evasion of anti-tumor immunity. Bladder cancer models in immunocompetent animals have been limited to the BBN chemical carcinogenesis [51] model and certain GEM models [8, 9, 11]. Compared with conventional models, our model is characterized by several advantages for experimental use. First, K5-mUrorganoids can be cryopreserved. Additionally, the in vivo tumor formation rate is consistently high, with a short and uniform time to tumor formation. A tumor can form at any location, such as subcutaneously, orthotopically, or renal subcapsularly. Furthermore, this system has a “clean” genetic background consisting of a small number of genetic mutations. In urothelial carcinoma, anti-PD-1/PD-L1 drugs have been adopted as the standard treatment approach [4, 27]. Modeling diseases and treatments using immunocompetent animals is essential for predicting the therapeutic effects of immunotherapy and elucidating resistance mechanisms. Our experimental results will undoubtedly greatly contribute to contemporary urothelial cancer research [6].

The PI3K/AKT/mTOR pathway is known as a critical factor for cancer metabolism, such as glycolysis (Warburg effect), the pentose phosphate pathway, fatty acid synthesis, tricarboxylic acid cycle, glutaminolysis, and OXPHOS. A clinically aggressive subgroup of non-invasive bladder cancer, which develops via a distinct molecular pathway from that of invasive bladder cancer [5], was reportedly characterized by aberrant activation of the PI3K/AKT/mTOR pathway and increased glycolysis [52]. However, the specific impacts of the PI3K/AKT/mTOR pathway on individual metabolic mechanisms are complicated and poorly understood, particularly in MIBC development. Here, we have demonstrated that *Pten* loss enriched expression of genes in the OXPHOS pathway and increased both the Mito and Glyco ATP generation rate. These findings are consistent with those of a previous report, which showed that the PI3K/AKT/mTOR pathway can positively regulate the OCR in head and neck cancer [53]. Another interesting fact is that the ROS pathway was also significantly upregulated on Pten loss, which potentially could be one of the factors to drive Trp53 LOH and tumorigenesis. This metabolic pathway may therefore be a promising target for new therapeutic strategies in MIBC, and our model may be a useful tool for investigating such approaches.

Our comparison of driver genes between human and mouse MIBC identified several KMT2-family genes with *KMT2C* as the most common gene between species. However, *Kmt2c* loss did not contribute to the tumor formation of K5-mUrorganoid. Since higher proliferation ability was shown in *Trp53*^*R172H/+*^ K5-mUrorganoid organoids deficient of both *Kmt2c* and *Pten* compared with those deficient of *Pten* alone, we assume that *Kmt2c* mutation may contribute to urothelial carcinogenesis in other way or context, which we hope to address in the future studies. According to the previous report, p53 is known to function as a co-activator of ASCOM [12]. Although this study did not directly investigate the role of p53 on ASCOM, it might be plausible that the p53 status could influence epigenetic regulation. Future studies using p53-chromatin immunoprecipitation along with an H3K4me profiling will be informative in the future.

We first generated organoids from Krt5-expressing mouse urothelial cells with the hypothesis that these are the originating cells of the human MIBC basal-squamous subtype. To trace the lineage, Krt5-expressing cell-specific Cre recombination was performed in vivo, followed by organoid generation and subsequent sorting for GFP+ cells. Because Krt5 is expressed across various organs in the body, generating a Krt5+ cell-derived autochthonous bladder cancer model was challenging. This approach led to the premature death of mice from other cancers developing before bladder cancer [11]. However, using an organoid culture system enabled the enrichment of bladder Krt5+ cells harboring the targeted genetic mutation, which helped overcome this problem.

We also previously attempted in vivo gene editing by injecting AAVs into mouse bladders, but the editing efficiency remained low and sufficient carcinogenesis was not achieved [16]. Conversely, organoids facilitated ex vivo gene editing using the CRISPR/Cas9 system and subsequent sorting allowed the aggregation of affected cells for transplantation back into the in vivo context. Consequently, these organoids represented a tumor with squamous differentiation that reflected intratumoral heterogeneity [54, 55]. In recent years, RNA expression-based subtype classification has become important for studying tumor immunity in bladder cancer [13, 20, 56–58]. The RNA expression patterns of the K5-mUrorganoid tumors corresponded to the basal-squamous subtype.

Organoids are a powerful tool for modeling the cancer initiation and progression processes [59–61]. In the ex vivo setting, organoid models offer an advantage of facile editing by applying CRISPR technology to investigate differences in phenotypes. Furthermore, a diverse range of assays can be feasibly performed using solely pure cancer cells, as demonstrated in this study. In the in vivo setting, obtaining uniformly sized tumors within 1–2 months using the syngeneic model can support drug testing and investigations into the tumor microenvironment, similarly to the established practices with cell lines [11]. Indeed, murine organ-derived organoids have been used for various types of cancer research to examine mechanisms of tumorigenesis [62], progression [63], and metastasis [41], although their application in urothelial cancer research has been limited [9].

In summary, we established syngeneic mouse models from organoids derived from Krt5-expressing bladder urothelial cells of GEM. *Trp53* LOH accompanied with a missense mutation and activation of the PI3K pathway were crucial factors for tumorigenesis. Given the high frequency of *p53* missense mutations and PI3K pathway activation in human MIBC, our K5-mUrorganoid model closely mimics the clinical scenarios observed with this disease. Therefore, our mouse models are expected to serve as potent experimental tools for cancer immunobiology research in the bladder cancer field.

## Materials and methods

Experimental procedures are provided in the Supplementary Materials and Methods.

## Supplementary information

Supplementary Figures and Table S1

Supplementary Materials and Methods

Supplementary Table S2

## Data Availability

RNA-seq, WGS and WES data from mouse samples have been deposited in the DNA Data Bank of Japan (DDBJ), under accession number BioProject: PRJDB17698 (PSUB022611), Run: DRR554984-DRR555065 (akihirohamada0110-0001_Run_0021-0102). WES data from human samples have been deposited at the Japanese Genotype-phenotype Archive (JGA, http://trace.ddbj.nig.ac.jp/jga), which is also hosted by the DDBJ, under accession number JGA S000748.
