# Supplementary material for: PTEN loss drives p53 LOH and immune evasion in a novel urothelial organoid model harboring p53 missense mutations
Source: Oncogene. 2025 Feb 22;44(19):1336–49. doi: 10.1038/s41388-025-03311-5 (PMC12052601; doi:10.1038/s41388-025-03311-5)
Supplement: Supplementary file 1 — Supplementary Figures and Table S1 [file 41388_2025_3311_MOESM1_ESM.pdf]

Supplementary information

PTEN Loss Drives p53 LOH and Immune Evasion in a Novel Urothelial Organoid Model Harboring p53 Missense Mutations

Hamada et al.

Figure S1

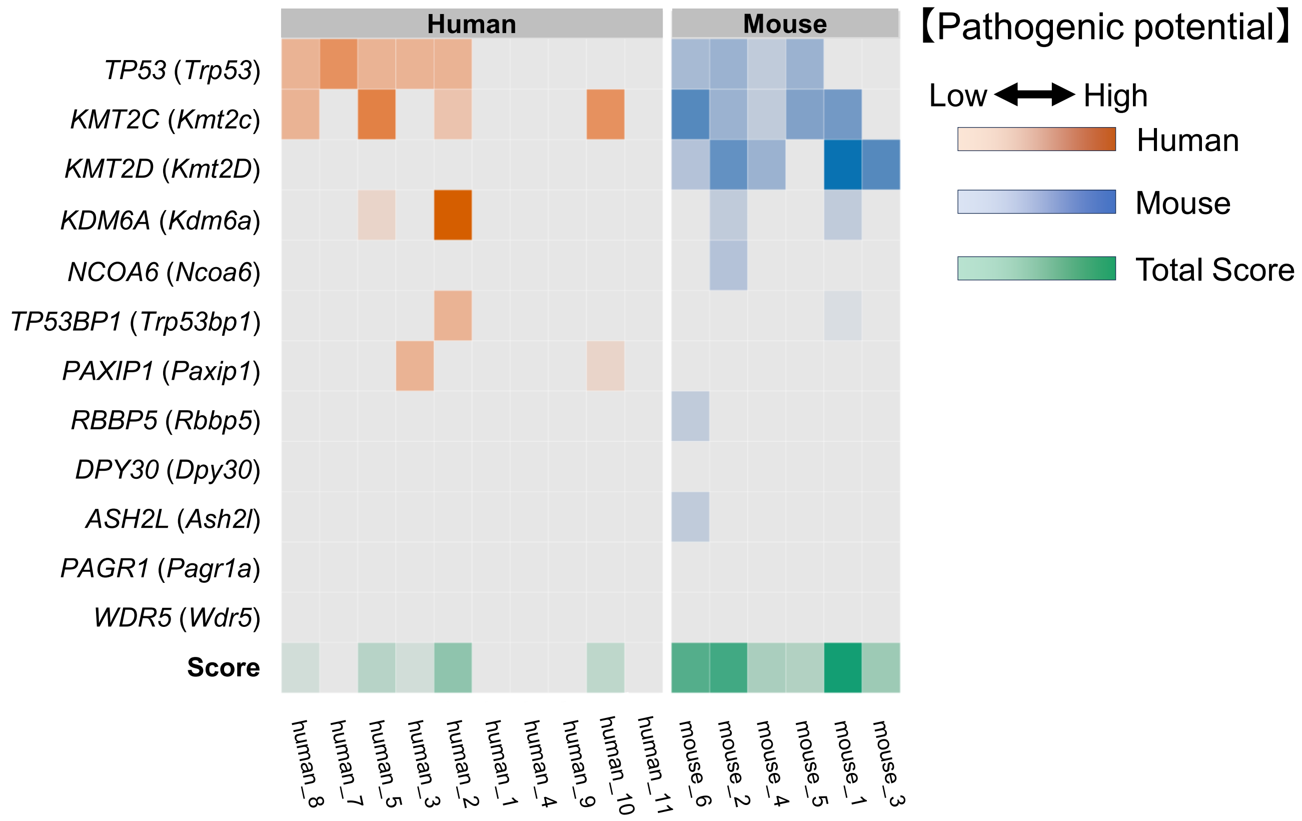

Supplementary Figure S1. Repeated *KMT2C* (*Kmt2c*) and *TP53* (*Trp53*) mutations were detected in both human and N-butyl-N-(4-hydroxybutyl)-nitrosamine (BBN)-induced mouse bladder cancer (related to Figure 1). Whole exome sequencing was performed on tumor tissues obtained from human muscle-invasive bladder cancer MIBC (n=10) and murine bladder cancer induced by BBN (n=6). Annotations of the pathogenic potential are represented by colored boxes using SnpEFF. Among genes involved in ASC-2/NCOA6 complex (ASCOM), a *KTM2C* (*Ktm2c*) alteration was most commonly observed in both human and murine bladder cancers.

Figure S2

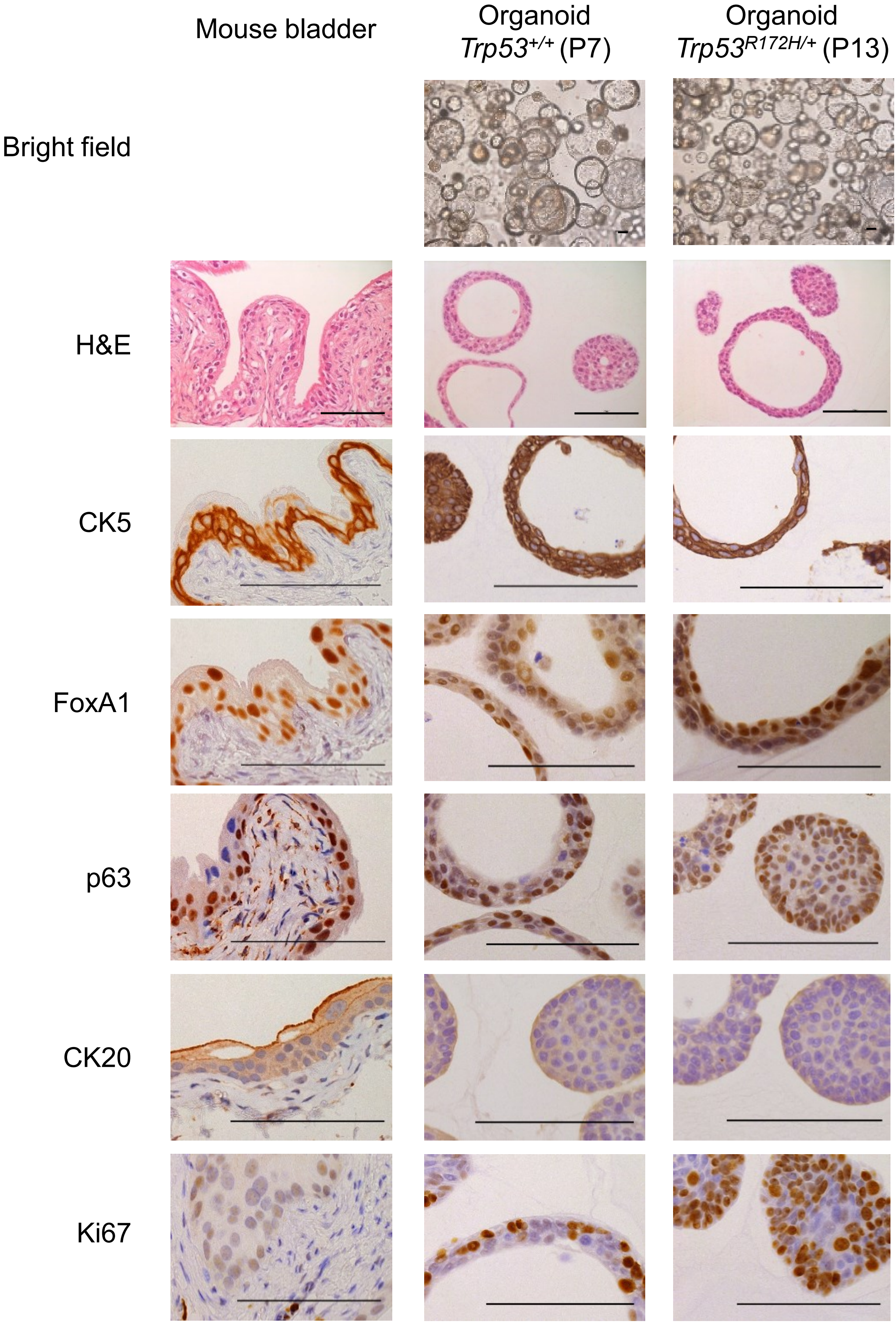
Mouse bladder
Organoid
Trp53+/+ (P7)
Organoid
Trp53R172H/+ (P13)
Bright field
H&E
CK5
FoxA1
p63
CK20
Ki67

Supplementary Figure S2. Histological features of normal mouse urothelium and organoids from genetically engineered mice (related to Figure 1). Representative microscopic images of normal mouse bladder urothelium (left), K5-mUrorganoids from *Trp53* wild-type (middle), and from *Trp53*$^{R172H/+}$ (right) mouse urothelium. Bars indicate 100 μm.

Figure S3

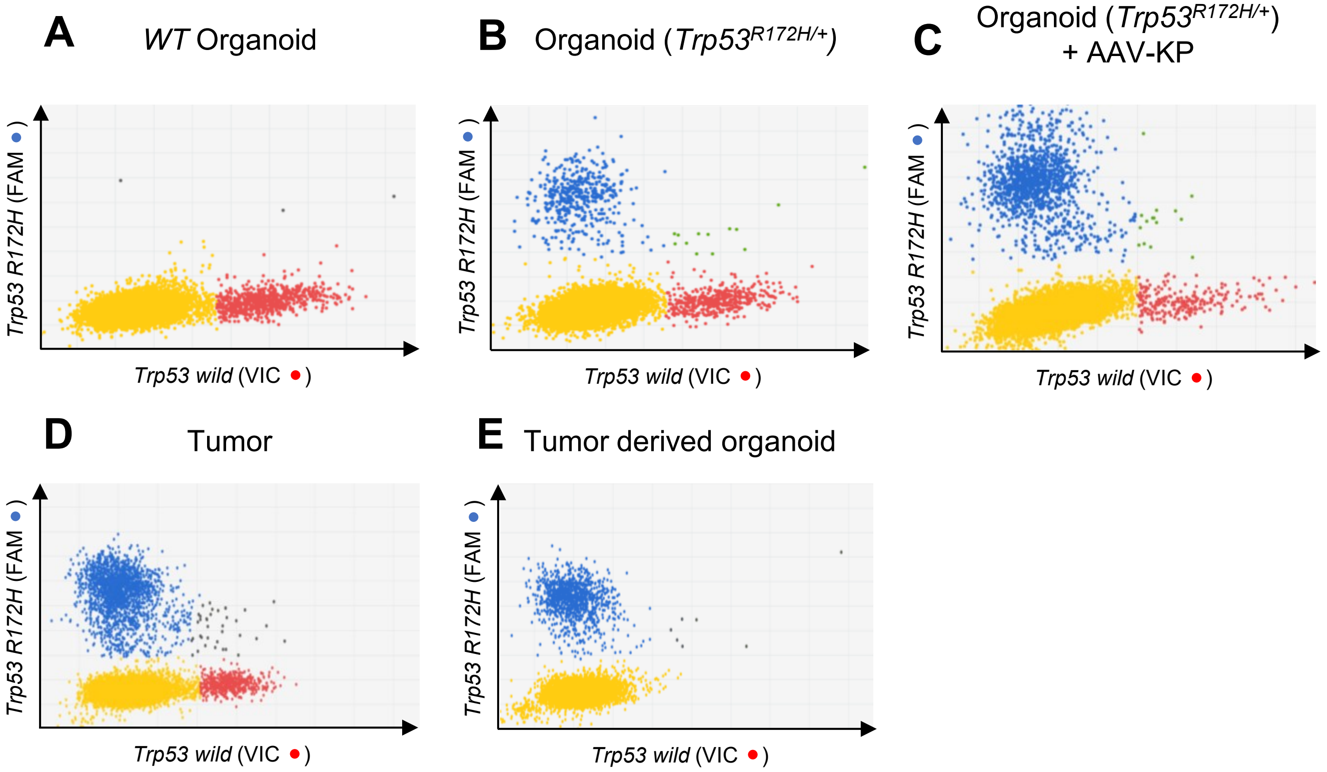

Supplementary Figure S3 (related to Figure 1). Representative images of digital PCR data for untreated **(A)** wild-type (WT), **(B)** *Trp53*$^{R172H/+}$ K5-mUrorganoids, **(C)** *Trp53*$^{R172H/+}$ K5-mUrorganoids treated with AAV-sgKmt2c-sgPten (AAV-KP), **(D)** tumors from *Trp53*$^{R172H/+}$ K5-mUrorganoids treated with AAV-KP, and **(E)** organoids derived from tumors from *Trp53*$^{R172H/+}$ K5-mUrorganoids treated with AAV-KP. The horizontal axes represent the WT allele (VIC), while the vertical axes represent the R172H (mutant) allele (FAM).

Figure S4

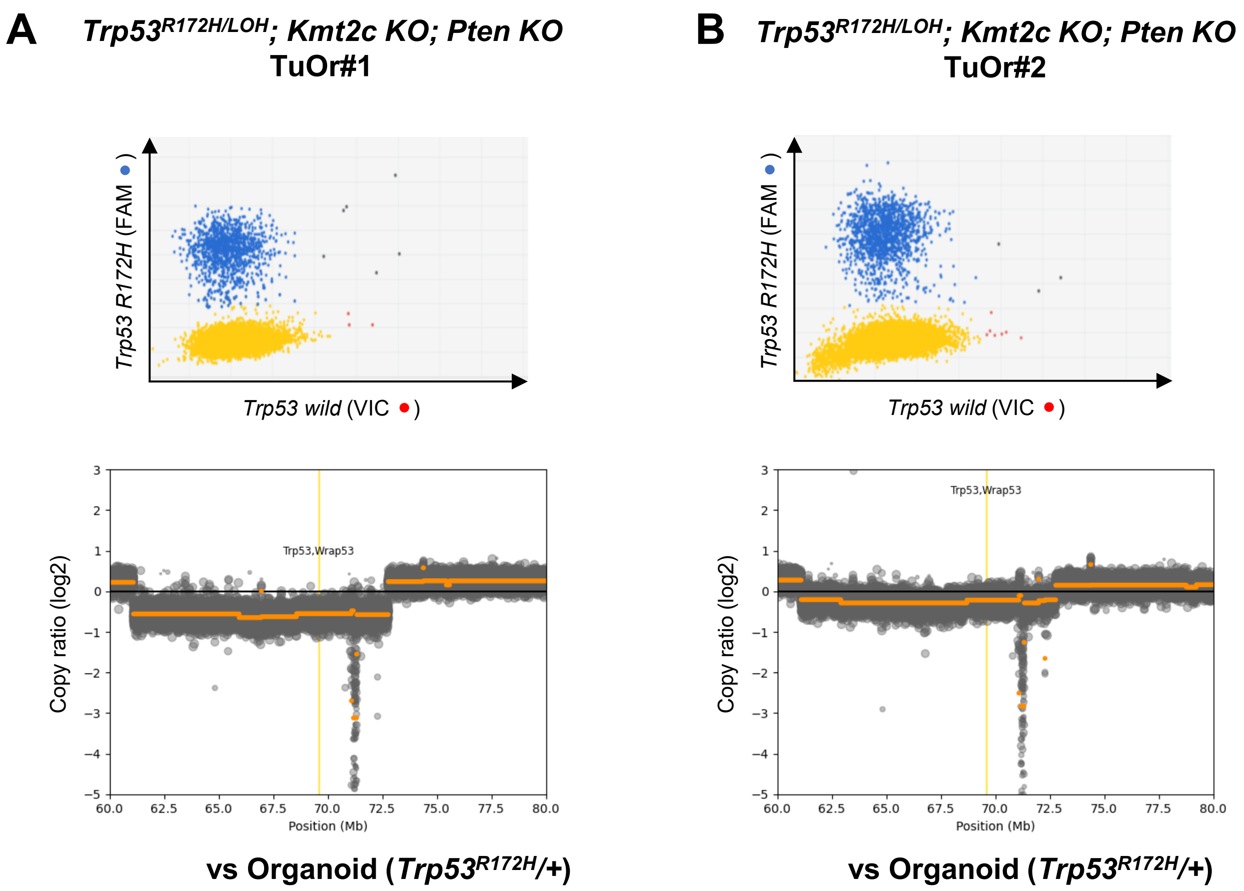

Supplementary Figure S4. Cells harboring *Trp53* mutant loss of heterozygosity (LOH) with copy number loss are enriched during the tumorigenic process (related to Figure 1). Digital PCR (top) and copy number analysis using whole genome sequencing (WGS, bottom) showing that cells harboring *Trp53*^{R172H} LOH with copy number loss became dominant in tumor-derived organoids (TuOrs) from two independent comparisons (**A**; TuOr#1, **B**; TuOr#2) with the original genotype of *Trp53*$^{R172H/+}$*; Kmt2c KO; Pten KO*. The yellow lines in the copy number analysis by WGS indicate the region where *Trp53* is coded.

Figure S5

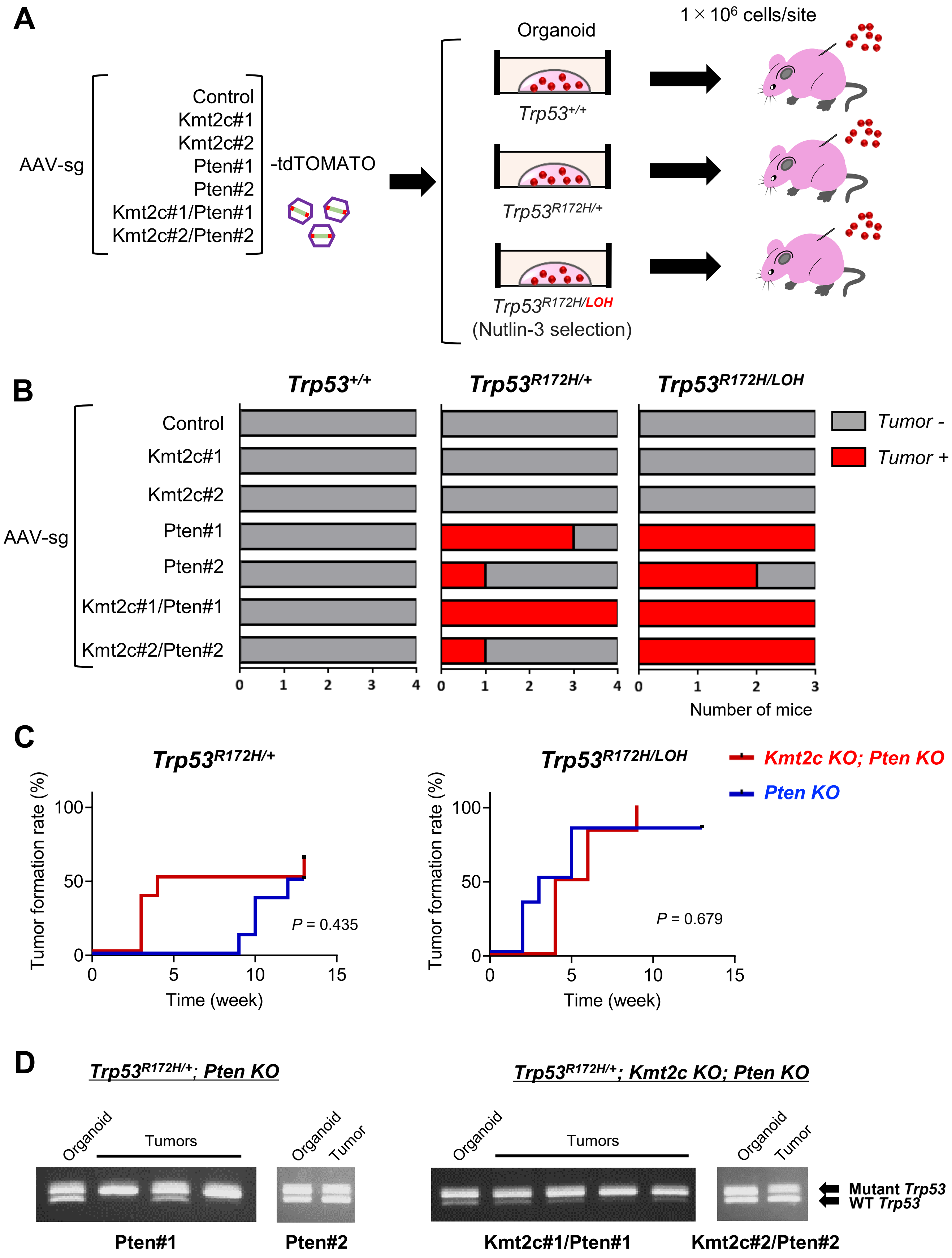
A
AAV-sg
Control
Kmt2c#1
Kmt2c#2
Pten#1
Pten#2
Kmt2c#1/Pten#1
Kmt2c#2/Pten#2
-tdTOMATO
Organoid
Trp53+/+
Trp53R172H/+
Trp53R172H/LOH
(Nutlin-3 selection)
Dissociated cells
1 × 10^6 cells/site
B
Trp53+/+
Trp53R172H/+
Trp53R172H/LOH
Tumor -
Tumor +
Number of mice
C
Trp53R172H/+
Trp53R172H/LOH
Kmt2c KO; Pten KO
Pten KO
Tumor formation rate (%)
Time (week)
P = 0.435
P = 0.679
D
Trp53R172H/+; Pten KO
Trp53R172H/+; Kmt2c KO; Pten KO
Organoid
Tumors
Tumor
Mutant Trp53
WT Trp53
Pten#1
Pten#2
Kmt2c#1/Pten#1
Kmt2c#2/Pten#2

Supplementary Figure S5. CRISPR/Cas9-based deletion of *Pten*, but not of *Ktm2c*, confers tumorigenic ability to *Trp53*-mutated K5-mUrorganoids (related to Figure 1). **A.** Schematics of the combination of single-guide (sg)RNA and K5-mUrorganoid genotype. *Trp53*$^{R172H/LOH}$ K5-mUrorganoids were generated by nutlin-3 selection. *Trp53* wild-type, *R172H/+,* and *R172H/LOH* K5-mUrorganoids with *Kmt2c* knockout (KO) and/or *Pten* KO were inoculated subcutaneously into athymic mice (n=3 to 4). **B.** Tumor formation rates with each combination of sgRNA and K5-mUrorganoid genotype. **C.** Kaplan–Meier curves for the time to tumor formation in *Trp53 R172H/+* (left) and *R172H/LOH* (right) K5-mUrorganoids with *Pten* KO alone (blue) vs. *Kmt2c* KO plus *Pten* KO (red). **D.** Representative results of genomic PCR for *Trp53* status in parent organoids and tumors with *Trp53*$^{R172H/+}$*; Pten KO* (left) and *Trp53*$^{R172H/+}$*; Kmt2c KO; Pten KO* (right).

Figure S6

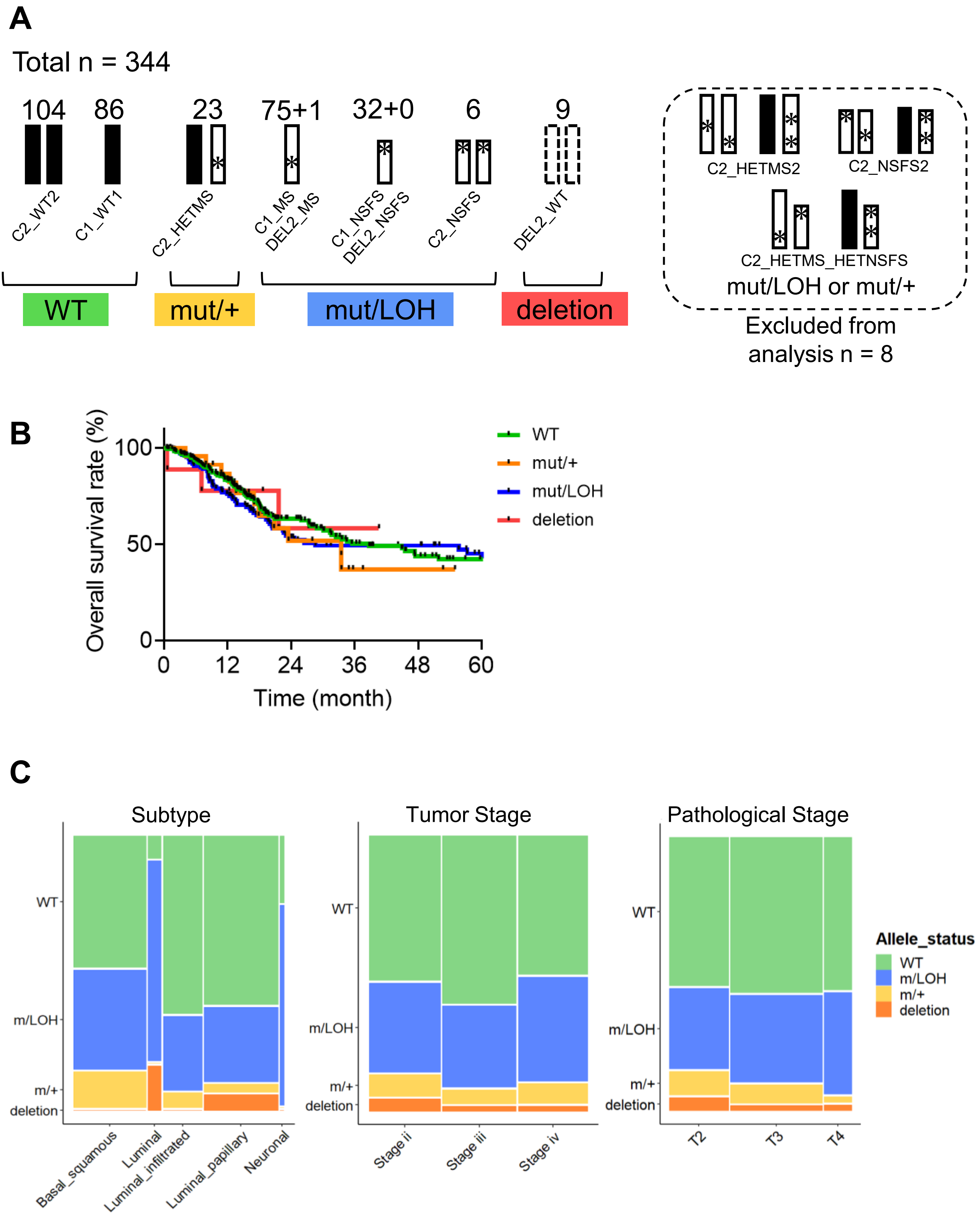
A
Total n = 344
104
86
23
75+1
32+0
6
9
C2_WT2
C1_WT1
C2_HETMS
C1_MS
DEL2_MS
C1_NSFS
DEL2_NSFS
C2_NSFS
DEL2_WT
WT
mut/+
mut/LOH
deletion
C2_HETMS2
C2_NSFS2
C2_HETMS_HETNSFS
mut/LOH or mut/+
Excluded from analysis n = 8
B
Overall survival rate (%)
100
50
0
0
12
24
36
48
60
Time (month)
WT
mut/+
mut/LOH
deletion
C
Subtype
Tumor Stage
Pathological Stage
WT
m/LOH
m/+
deletion
Basal_squamous
Luminal
Luminal_infiltrated
Luminal_papillary
Neuronal
Stage ii
Stage iii
Stage iv
T2
T3
T4
Allele_status
WT
m/LOH
m/+
deletion

Supplementary Figure S6. The Cancer Genome Atlas (TCGA) data analysis of *p53* status (related to Figure 2). A. Overall, 344 patients were included in the analysis. *P53* status was assigned according to the mutation call count and copy number. Eight patients were excluded from the analysis because of the possibility of more than one genotype. **B.** Overall survival rate analysis regarding the p53 genotype in TCGA data. **C.** Distribution of the patients with each *p53* status according to the molecular subtype (left), tumor stage (middle), and pathological stage (right).

Figure S7

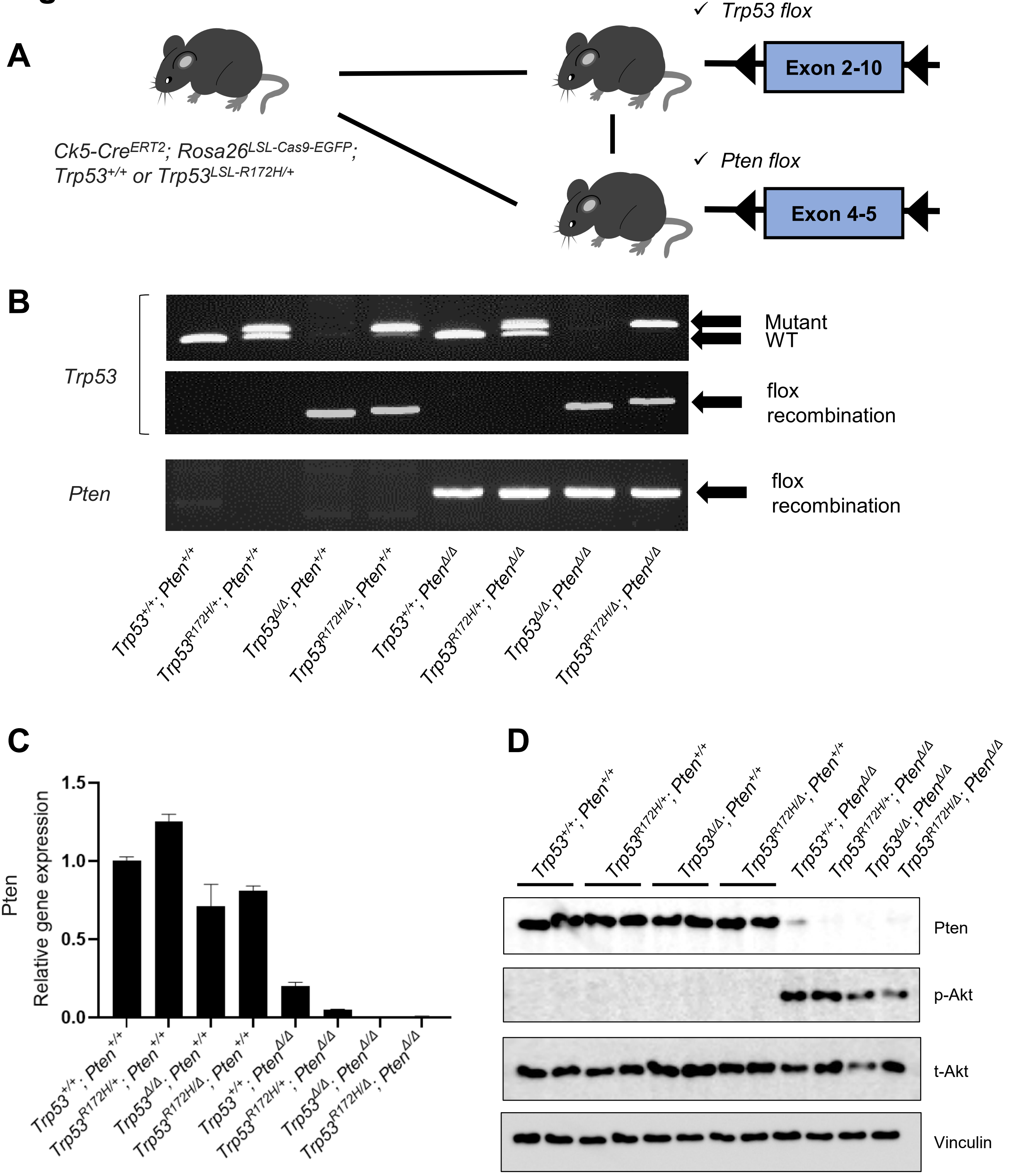
A
✓ Trp53 flox
Exon 2-10
✓ Pten flox
Exon 4-5
Ck5-CreERT2; Rosa26LSL-Cas9-EGFP;
Trp53+/+ or Trp53LSL-R172H/+
B
Trp53
Mutant
WT
flox recombination
Pten
flox recombination
Trp53+/+; Pten+/+
Trp53R172H/+; Pten+/+
Trp53Δ/Δ; Pten+/+
Trp53R172H/Δ; Pten+/+
Trp53+/+; PtenΔ/Δ
Trp53R172H/+; PtenΔ/Δ
Trp53Δ/Δ; PtenΔ/Δ
Trp53R172H/Δ; PtenΔ/Δ
C
Pten
Relative gene expression
1.5
1.0
0.5
0.0
Trp53+/+; Pten+/+
Trp53R172H/+; Pten+/+
Trp53Δ/Δ; Pten+/+
Trp53R172H/Δ; Pten+/+
Trp53+/+; PtenΔ/Δ
Trp53R172H/+; PtenΔ/Δ
Trp53Δ/Δ; PtenΔ/Δ
Trp53R172H/Δ; PtenΔ/Δ
D
Trp53+/+; Pten+/+
Trp53R172H/+; Pten+/+
Trp53Δ/Δ; Pten+/+
Trp53R172H/Δ; Pten+/+
Trp53+/+; PtenΔ/Δ
Trp53R172H/+; PtenΔ/Δ
Trp53Δ/Δ; PtenΔ/Δ
Trp53R172H/Δ; PtenΔ/Δ
Pten
p-Akt
t-Akt
Vinculin

Supplementary Figure S7. Genotyping of *Trp53* flox and *Pten* flox mice (related to Figure 2). A. Crossing schema of genetically engineered mice. **B.** Genotyping PCR of the eight K5-mUrorganoid strains. **C.** Relative Pten mRNA expression of the eight K5-mUrorganoid strains by quantitative real-time PCR. **D.** Western blot analysis of the eight K5- mUrorganoid strains.

Figure S8

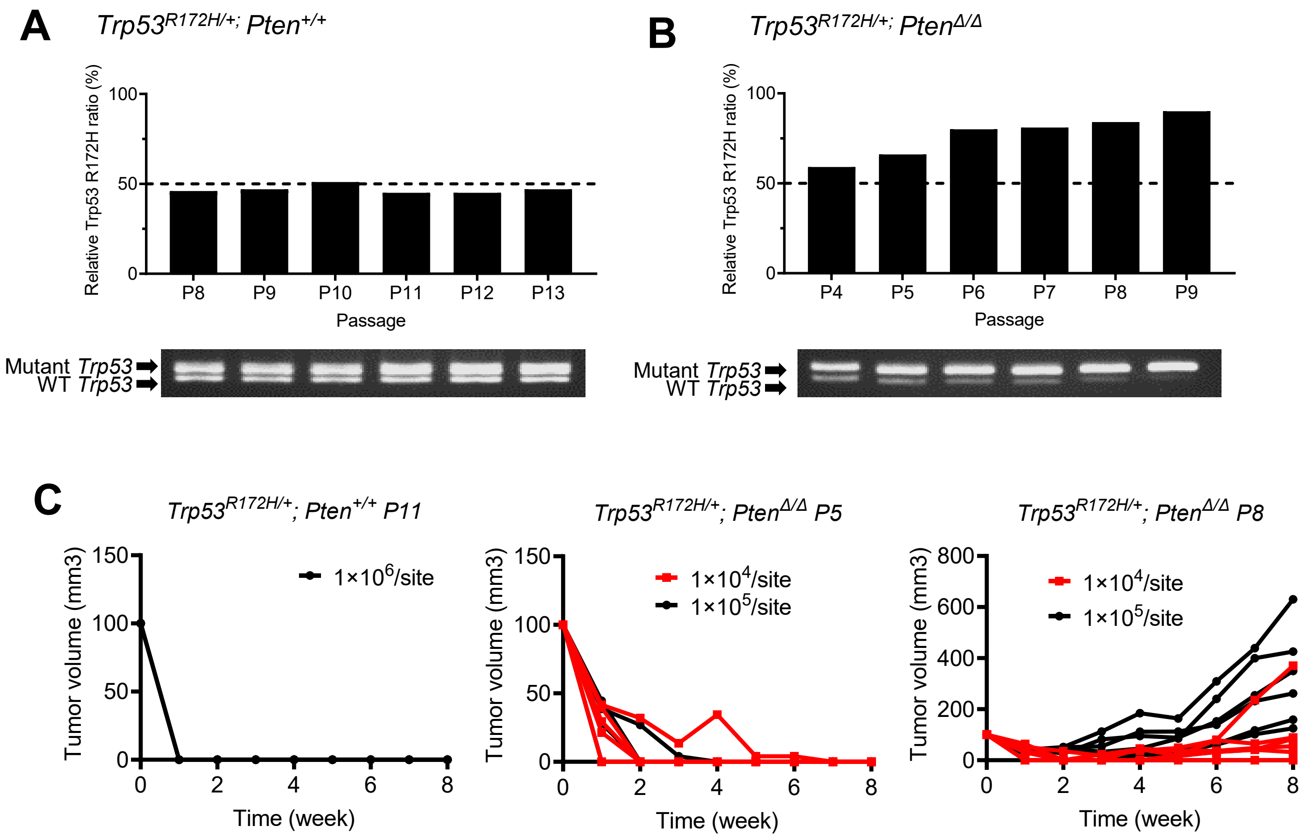

Supplementary Figure S8. K5-mUrorganoids with *Trp53* heterozygous mutation and *Pten* deletion increases the mutant allele ratio through passaging and can gain a tumor-forming ability (related to Figure 2). A-B. The ratio of *Trp53 R172H* to wild-type allele in **(A)** *Trp53$^{R172H/+;}$ Pten$^{+/+}$* and **(B)** *Trp53$^{R172H/+;}$ Pten$^{Δ/Δ}$* K5-mUrorganoids through passaging was determined by digital PCR (top) and confirmed using gel electrophoresis bands after genomic PCR (bottom). **C.** Subcutaneous tumor formation ability in athymic mice of K5-mUrorganoids with the indicated genotype and passage number (P) are shown in the spider plots. Left; K5-mUrorganoids with *Trp53$^{R172H/+;}$ Pten$^{+/+}$* after 11 passages (P11, 1×10^6 cells/site, n=8). Middle; K5-mUrorganoids with *Trp53$^{R172H/+;}$ Pten$^{Δ/Δ}$* P5 (1×10^4 cells/site in red, 1×10^5 cells/site in black, n=6 each). Right; K5-mUrorganoids with *Trp53$^{R172H/+;}$ Pten$^{Δ/Δ}$* P8 (1×10^4 cells/site in red, 1×10^5 cells/site in black, n=6 each).

Figure S9

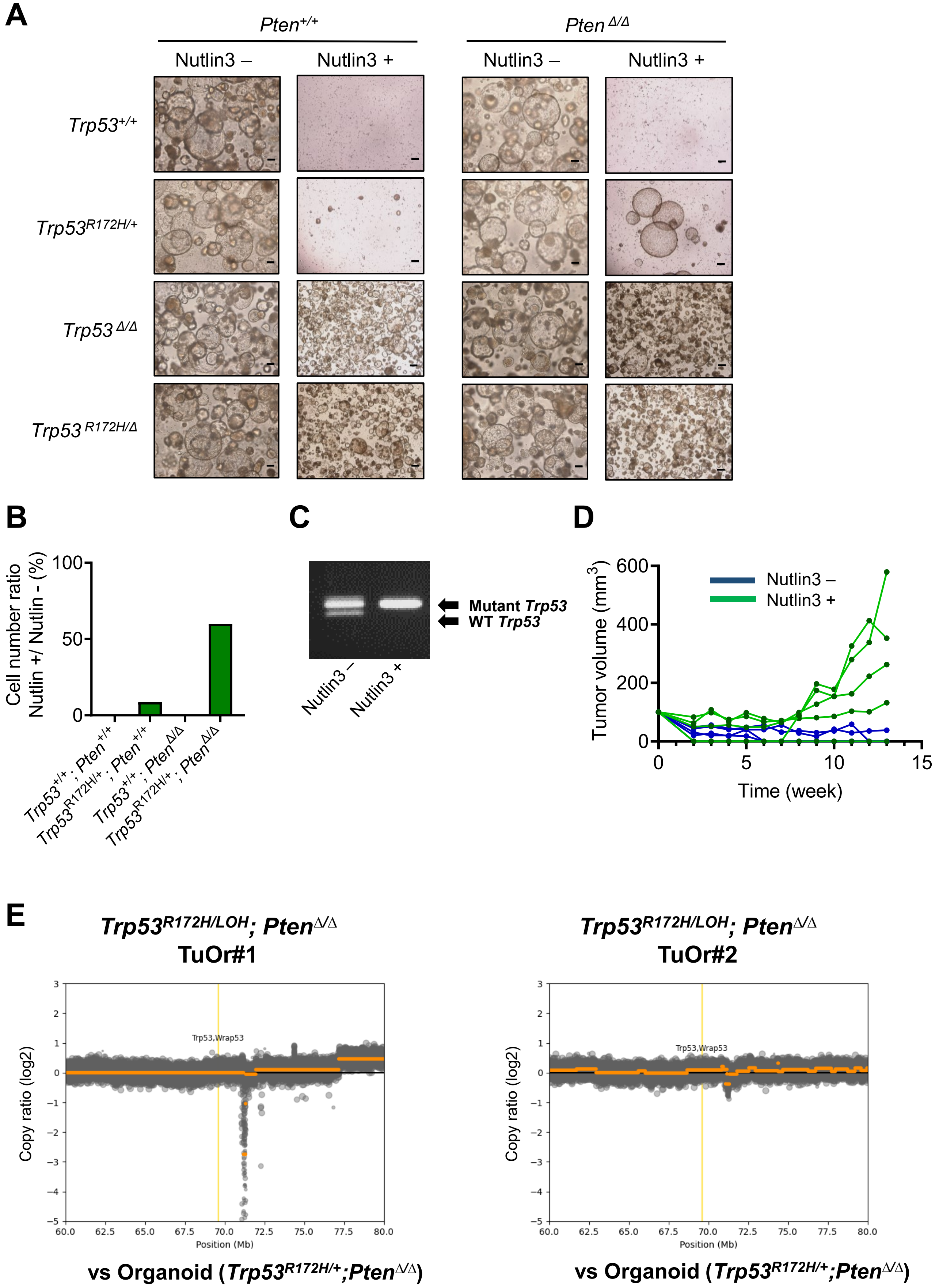

A
Pten+/+
PtenΔ/Δ
Nutlin3 –
Nutlin3 +
Trp53+/+
Trp53R172H/+
Trp53Δ/Δ
Trp53R172H/Δ
B
Cell number ratio
Nutlin +/ Nutlin - (%)
100
50
0
Trp53+/+ ; Pten+/+
Trp53R172H/+ ; Pten+/+
Trp53+/+ ; PtenΔ/Δ
Trp53R172H/+ ; PtenΔ/Δ
C
Mutant Trp53
WT Trp53
Nutlin3 –
Nutlin3 +
D
Tumor volume (mm3)
600
400
200
0
Nutlin3 –
Nutlin3 +
0
5
10
15
Time (week)
E
Trp53R172H/LOH; PtenΔ/Δ
TuOr#1
Trp53R172H/LOH; PtenΔ/Δ
TuOr#2
Copy ratio (log2)
Trp53,Wrap53
Position (Mb)
60.0
62.5
65.0
67.5
70.0
72.5
75.0
77.5
80.0
vs Organoid (Trp53R172H/+;PtenΔ/Δ)

Supplementary Figure S9. *Pten* loss promotes *Trp53* loss of heterozygosity (LOH) and subsequent tumorigenic ability (related to Figure 2). A. Representative blight-field images of K5-mUrorganoids harboring eight distinct genotypes in the absence (nutlin-3 –, left) and presence (nutlin-3 +, right) of the Murine double minute 2 (MDM2) inhibitor nutlin-3. **B.** The relative cell number of K5-mUrorganoids harboring the indicated genotypes cultured in the presence of nutlin-3 compared with those cultured in the absence of nutlin-3. **C.** PCR band for wild-type (WT) and mutant *Trp53* of $Trp53^{R172H/+;}$ $Pten^{\Delta/\Delta}$ K5-mUrorganoids cultured in the absence (nutlin-3 –) or presence (nutlin-3 +) of nutlin-3. **D.** Changes in tumor size after subcutaneous inoculation of $Trp53^{R172H/+;}$ $Pten^{\Delta/\Delta}$ K5-mUrorganoids cultured in the absence (blue) or presence (green) of nutlin-3 in athymic mice (1×10^6 cells/mouse, n=5 each). **E.** Tumor-derived organoids (TuOrs) were generated from two representative tumors developed from $Trp53^{R172H/+;}$ $Pten^{\Delta/\Delta}$ K5-mUrorganoids inoculated after nutlin-3 selection. These TuOrs showed copy number neutral LOH of mutant *Trp53* in the whole genome sequencing analysis.

Figure S10

A

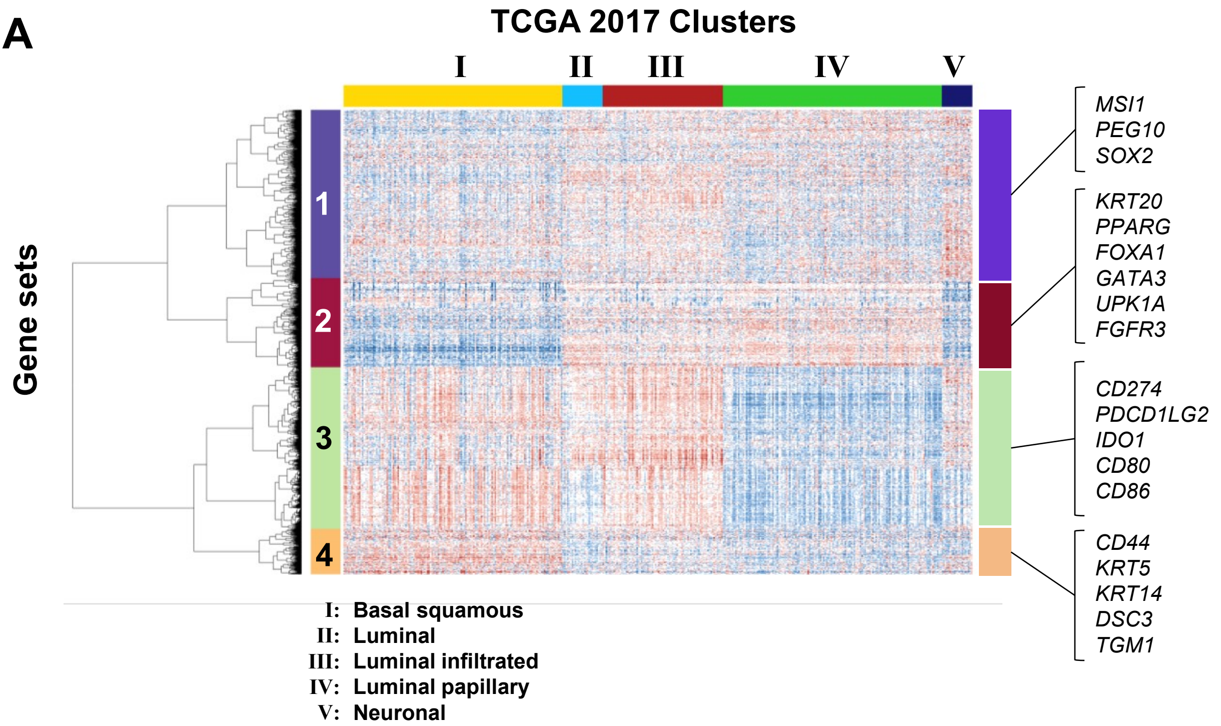

Modified from Masuda et al AM J Pathol 2020[15]

B

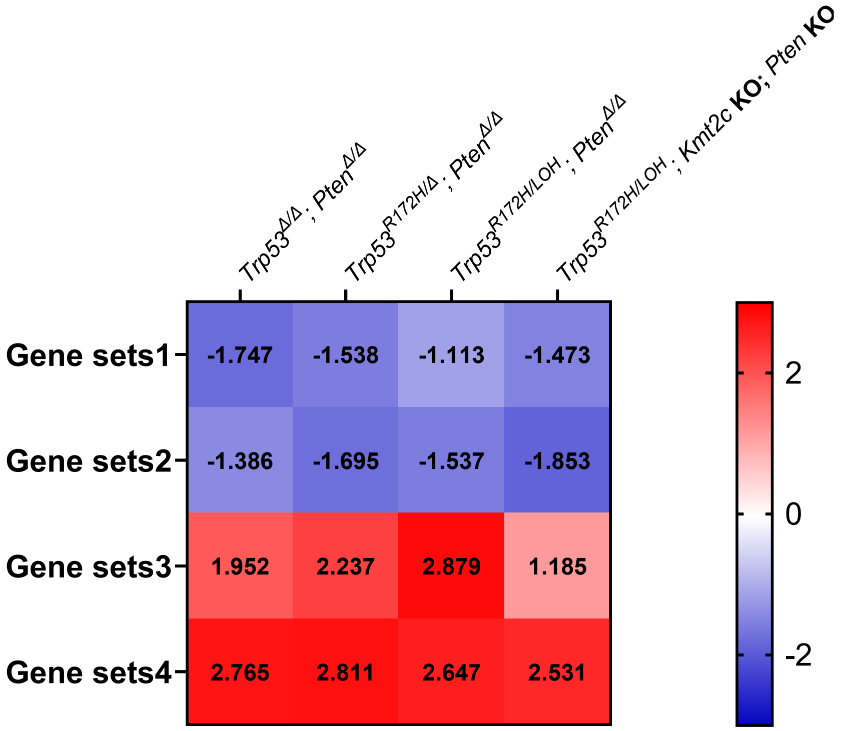

Supplementary Figure S10. Tumors harboring the molecular characteristics of the human muscle-invasive bladder cancer basal-squamous subtype (related to Figure 2). A. Hierarchical clustering of The Cancer Genome Atlas (TCGA) 2017 gene expression profiles identified four gene sets with distinct enrichment patterns for each TCGA 2017 cluster, which was modified from a previous report (15). **B.** A heat map with normalized enrichment scores (NES) in the gene set enrichment analysis (GSEA) of bladder tumors derived from K5-mUrorganoids with the indicated genotypes.

Figure S11

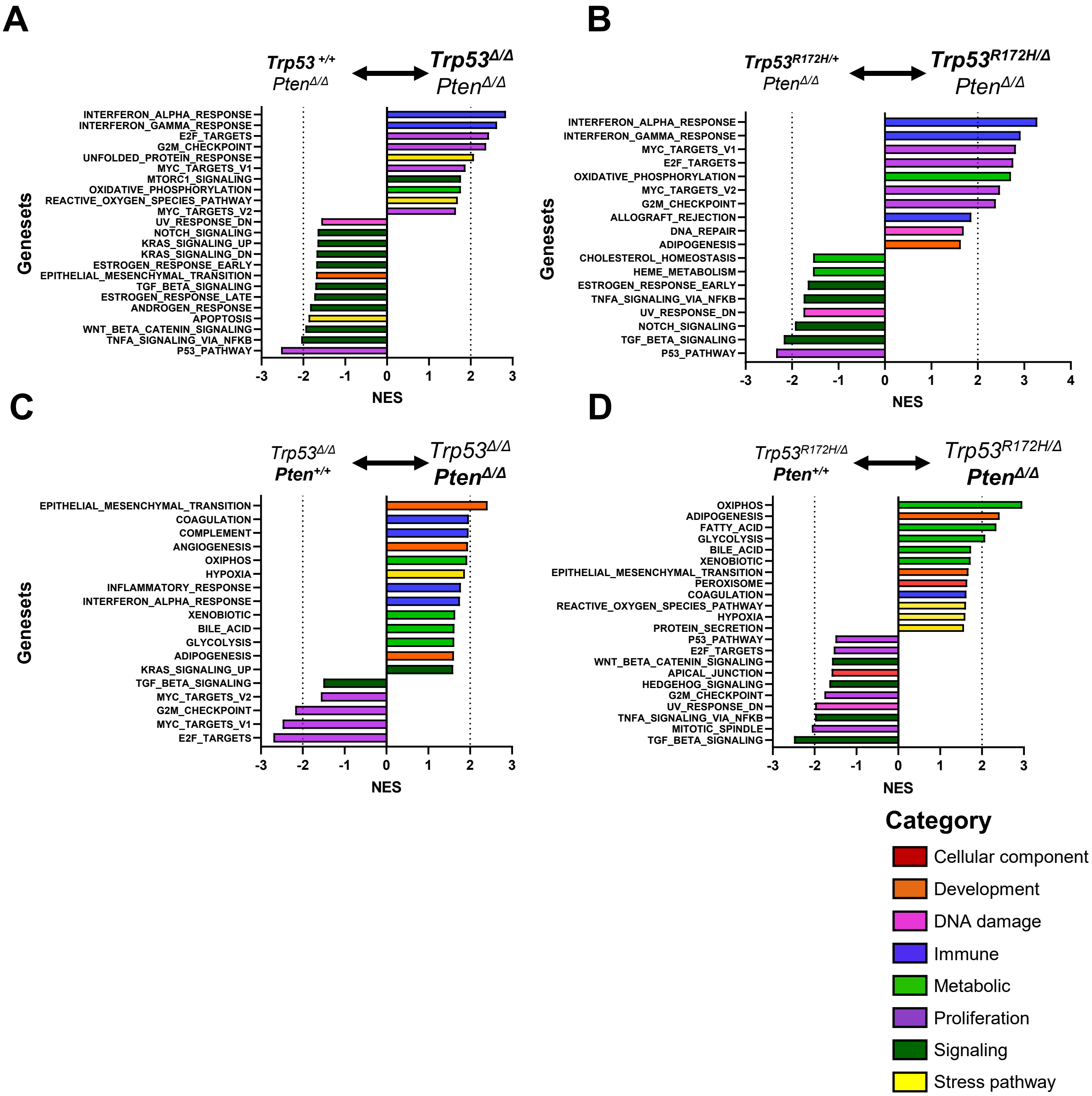

A
Trp53 +/+ PtenΔ/Δ
Trp53Δ/Δ PtenΔ/Δ
INTERFERON_ALPHA_RESPONSE
INTERFERON_GAMMA_RESPONSE
E2F_TARGETS
G2M_CHECKPOINT
UNFOLDED_PROTEIN_RESPONSE
MYC_TARGETS_V1
MTORC1_SIGNALING
OXIDATIVE_PHOSPHORYLATION
REACTIVE_OXYGEN_SPECIES_PATHWAY
MYC_TARGETS_V2
UV_RESPONSE_DN
NOTCH_SIGNALING
KRAS_SIGNALING_UP
KRAS_SIGNALING_DN
ESTROGEN_RESPONSE_EARLY
EPITHELIAL_MESENCHYMAL_TRANSITION
TGF_BETA_SIGNALING
ESTROGEN_RESPONSE_LATE
ANDROGEN_RESPONSE
APOPTOSIS
WNT_BETA_CATENIN_SIGNALING
TNFA_SIGNALING_VIA_NFKB
P53_PATHWAY
Genesets
NES
B
Trp53R172H/+ PtenΔ/Δ
Trp53R172H/Δ PtenΔ/Δ
INTERFERON_ALPHA_RESPONSE
INTERFERON_GAMMA_RESPONSE
MYC_TARGETS_V1
E2F_TARGETS
OXIDATIVE_PHOSPHORYLATION
MYC_TARGETS_V2
G2M_CHECKPOINT
ALLOGRAFT_REJECTION
DNA_REPAIR
ADIPOGENESIS
CHOLESTEROL_HOMEOSTASIS
HEME_METABOLISM
ESTROGEN_RESPONSE_EARLY
TNFA_SIGNALING_VIA_NFKB
UV_RESPONSE_DN
NOTCH_SIGNALING
TGF_BETA_SIGNALING
P53_PATHWAY
Genesets
NES
C
Trp53Δ/Δ Pten+/+
Trp53Δ/Δ PtenΔ/Δ
EPITHELIAL_MESENCHYMAL_TRANSITION
COAGULATION
COMPLEMENT
ANGIOGENESIS
OXIPHOS
HYPOXIA
INFLAMMATORY_RESPONSE
INTERFERON_ALPHA_RESPONSE
XENOBIOTIC
BILE_ACID
GLYCOLYSIS
ADIPOGENESIS
KRAS_SIGNALING_UP
TGF_BETA_SIGNALING
MYC_TARGETS_V2
G2M_CHECKPOINT
MYC_TARGETS_V1
E2F_TARGETS
Genesets
NES
D
Trp53R172H/Δ Pten+/+
Trp53R172H/Δ PtenΔ/Δ
OXIPHOS
ADIPOGENESIS
FATTY_ACID
GLYCOLYSIS
BILE_ACID
XENOBIOTIC
EPITHELIAL_MESENCHYMAL_TRANSITION
PEROXISOME
COAGULATION
REACTIVE_OXYGEN_SPECIES_PATHWAY
HYPOXIA
PROTEIN_SECRETION
P53_PATHWAY
E2F_TARGETS
WNT_BETA_CATENIN_SIGNALING
APICAL_JUNCTION
HEDGEHOG_SIGNALING
G2M_CHECKPOINT
UV_RESPONSE_DN
TNFA_SIGNALING_VIA_NFKB
MITOTIC_SPINDLE
TGF_BETA_SIGNALING
NES
Category
Cellular component
Development
DNA damage
Immune
Metabolic
Proliferation
Signaling
Stress pathway

Supplementary Figure S11. Gene set enrichment analysis (GSEA) showed distinct changes in biological pathways enriched by *Pten* loss and wild-type *Trp53* loss, respectively (related to Figure 3). Bar charts showing hallmark gene sets that were positively or negatively enriched between the indicated genotypes by normalized enrichment score (NES) >1.5 or <-1.5. Each gene set is color-coded by the indicated categories. **A.** $Trp53^{\Delta/\Delta}$; $Pten^{\Delta/\Delta}$ vs. $Trp53^{+/+}$; $Pten^{\Delta/\Delta}$. **B.** $Trp53^{R172H/\Delta}$; $Pten^{\Delta/\Delta}$ vs. $Trp53^{R172H/+}$; $Pten^{\Delta/\Delta}$. **C.** $Trp53^{\Delta/\Delta}$; $Pten^{\Delta/\Delta}$ vs. $Trp53^{\Delta/\Delta}$; $Pten^{+/+}$. **D.** $Trp53^{R172H/\Delta}$; $Pten^{\Delta/\Delta}$ vs. $Trp53^{R172H/\Delta}$; $Pten^{+/+}$.

Figure S12

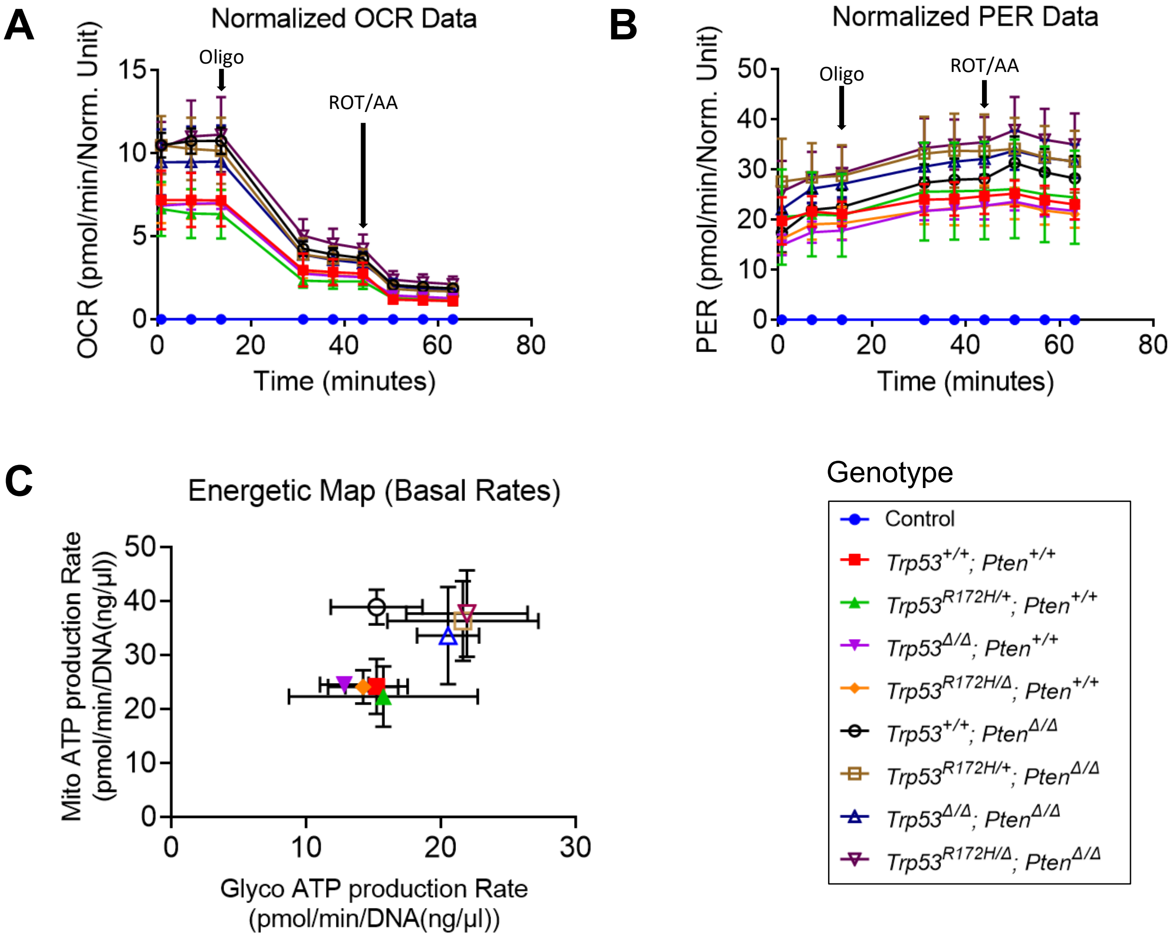

Supplementary Figure S12. Seahorse ATP assay showing that the ATP production rates from both mitochondrial respiration and glycolysis were higher in K5-mUrorganoids with *Pten* loss regardless of *Trp53* status (related to Figure 3). A. Mitochondrial respiration rates are shown as the normalized oxygen consumption rate (OCR). **B.** Glycolysis rates are shown as the normalized proton efflux rate (PER). **C.** Energy Map (basal rates) with the ATP production rate from glycolysis (x-axis) and the ATP production rate from mitochondrial respiration (y-axis).

Figure S13

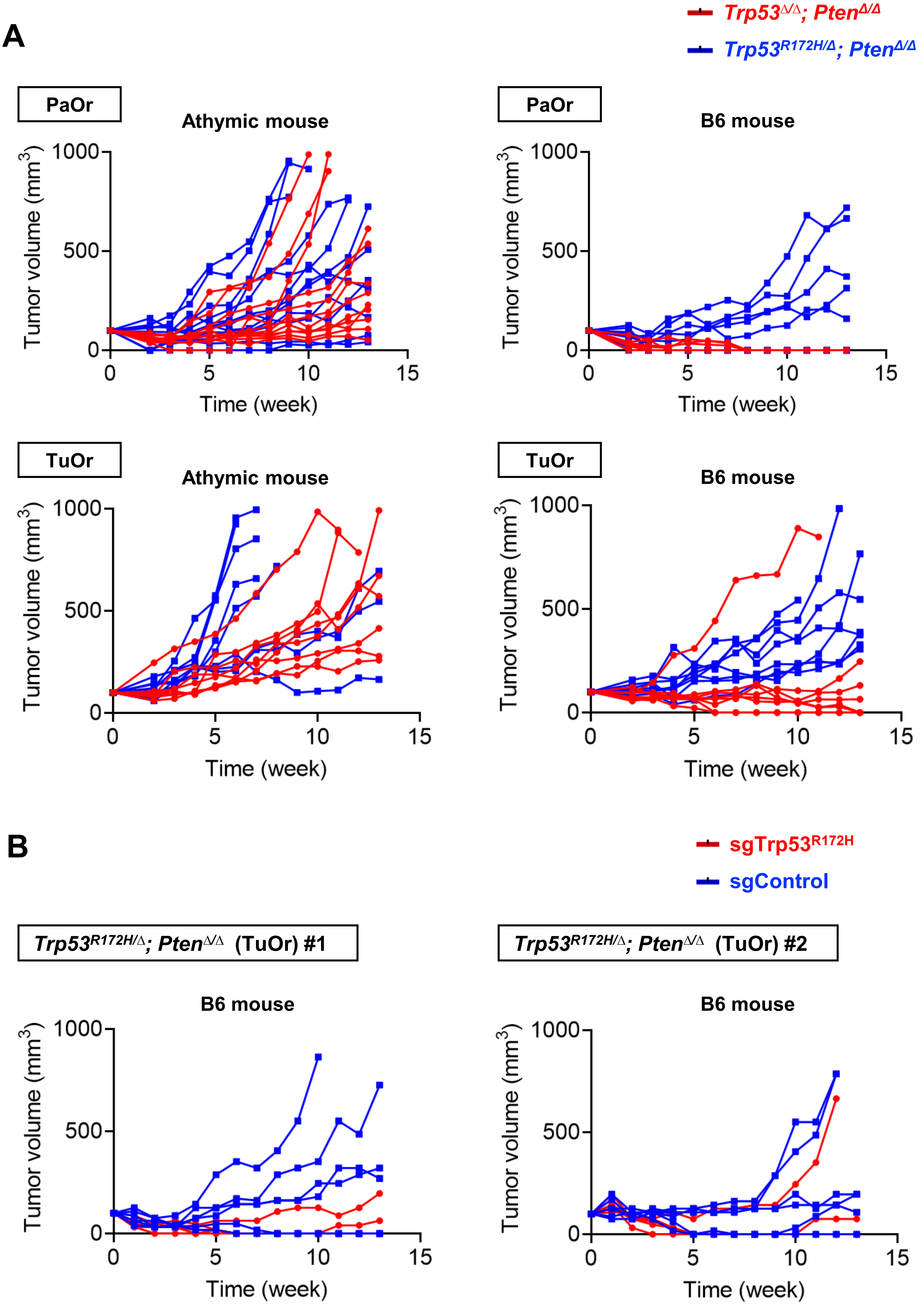
A
Trp53Δ/Δ; PtenΔ/Δ
Trp53R172H/Δ; PtenΔ/Δ
PaOr
Athymic mouse
PaOr
B6 mouse
TuOr
Athymic mouse
TuOr
B6 mouse
Tumor volume (mm3)
Time (week)
0
500
1000
0
5
10
15
B
sgTrp53R172H
sgControl
Trp53R172H/Δ; PtenΔ/Δ (TuOr) #1
Trp53R172H/Δ; PtenΔ/Δ (TuOr) #2
B6 mouse
B6 mouse

Supplementary Figure S13. *In vivo* tumor size changes (related to Figure 4). A. Here, 1×10^6 cells of parent (PaOr) and tumor-derived (TuOr) K5-mUrorganoids with the indicated genotypes were inoculated into the flank subcutaneous space of athymic (n=12) or immunocompetent B6 (n=8) mice. The size changes in each tumor are shown in the spider plots. B. Two independent *Trp53*$^{R172H/\Delta}$*; Pten*$^{\Delta/\Delta}$ TuOrs were infected with AAV-sgTrp53^{R172H} or AAV-sgControl. Then, 1×10^6 cells of each TuOr were inoculated subcutaneously into immunocompetent mice (n=6 each). The size changes or individual tumors are shown in the spider plots.

Figure S14

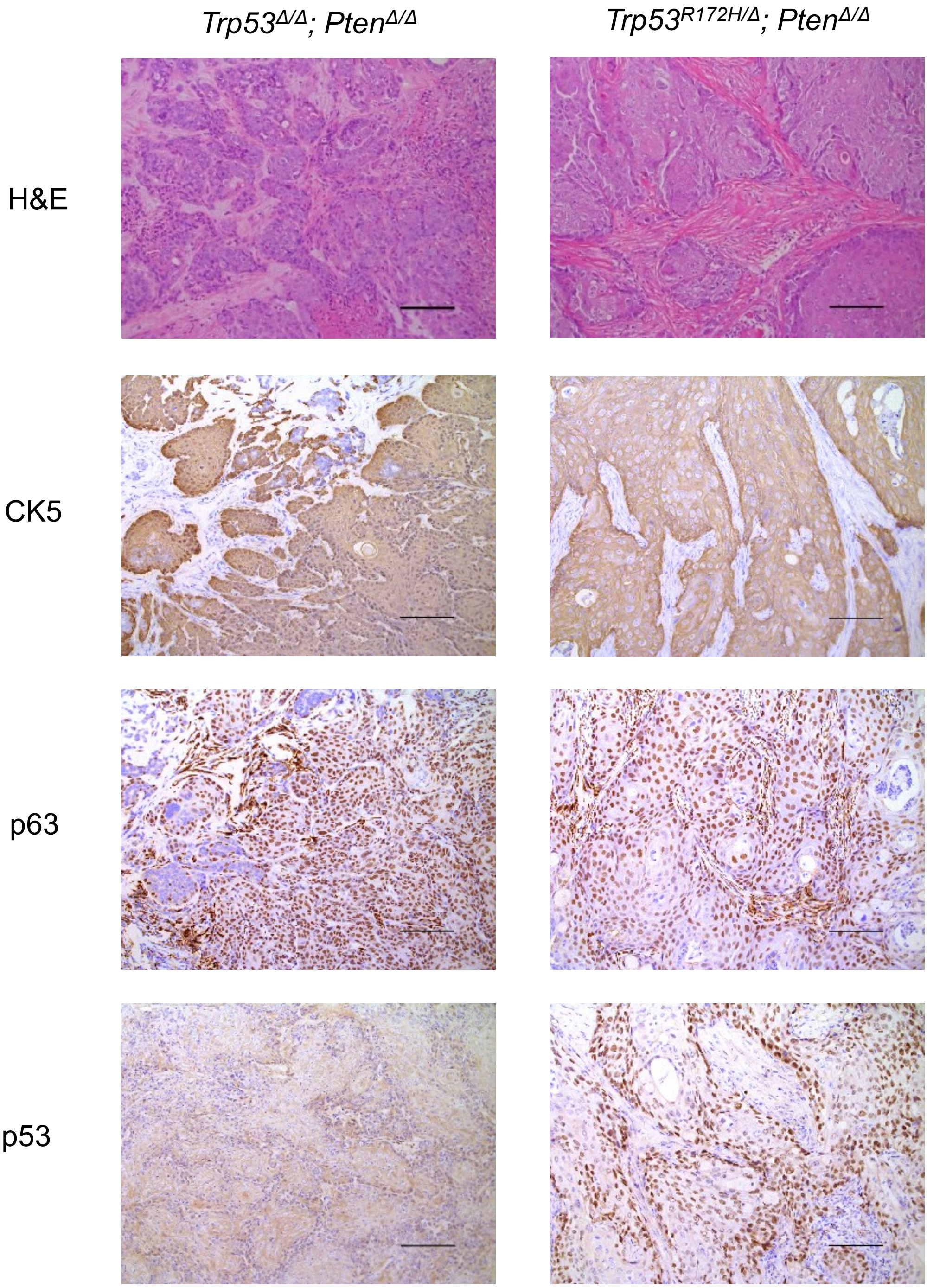

Supplementary Figure S14. Representative histological photomicrographs of tumors from tumor-derived *$Trp53^{\Delta/\Delta}$; $Pten^{\Delta/\Delta}$* and *$Trp53^{R172H/\Delta}$; $Pten^{\Delta/\Delta}$* K5-mUrorganoids (related to Figure 4). Hematoxylin and eosin (H&E) staining and immunohistochemistry assays of the indicated basal subtype markers were performed. Scale bars indicate 100 µm.

Figure S15

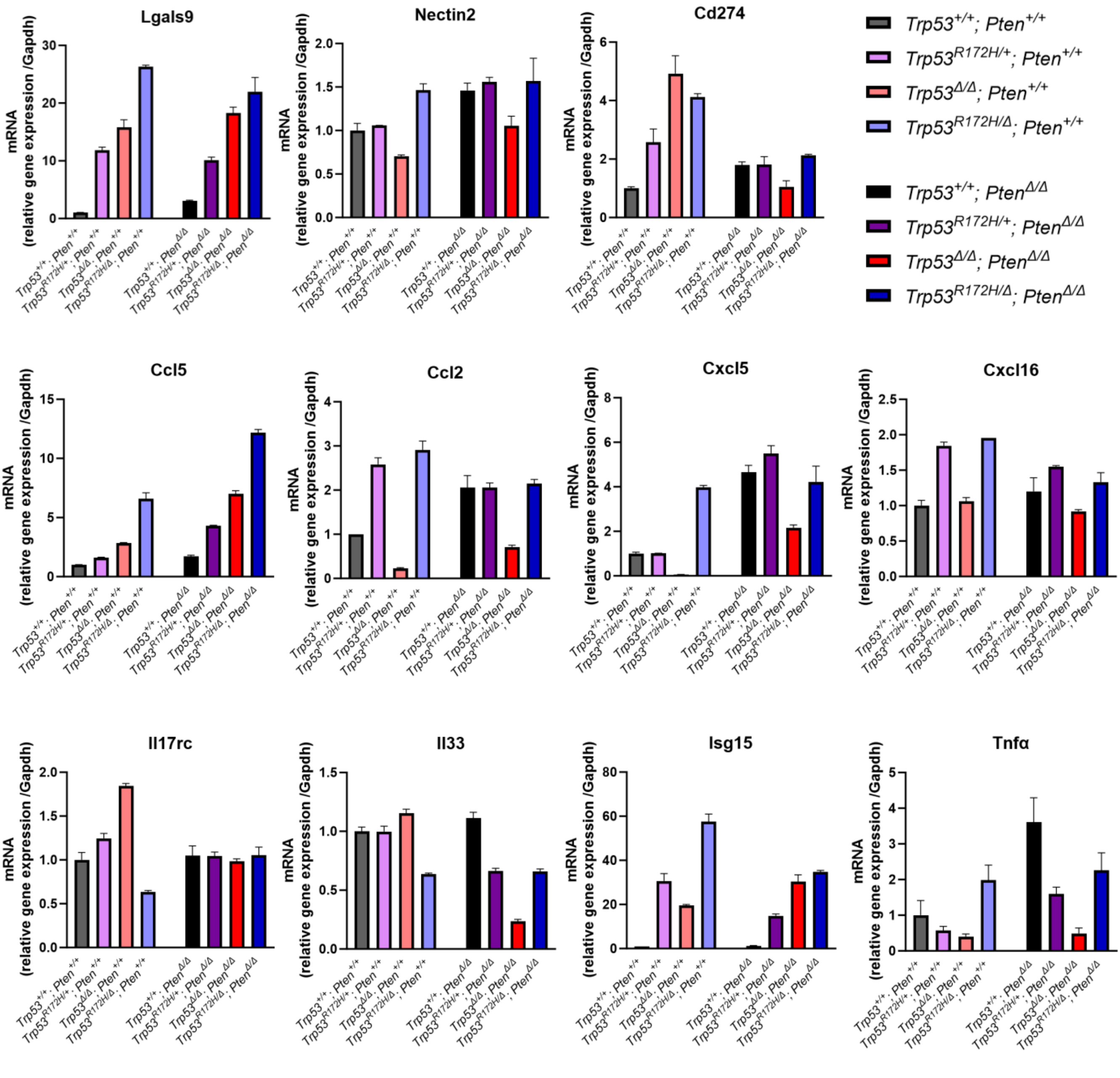

Supplementary Figure S15. Some of the genes related to tumor immune microenvironment including *Lgals9* and *Ccl5* were differentially expressed according to *Trp53* status, but regardless of *Pten* status (related to Figure 6). Validation of immune checkpoint related genes, cytokines and chemokines RNA expression for the eight parent K5-mUrorganoid strains by quantitative real-time PCR.

Table S1

Mouse Genotyping primer		
**Gene**	**Direction**	**Sequence 5' → 3'**
Cas9 Mutant	FWD	TGAGCGACATCCTGAGAGTG
	REV	GAGAGCTTTCAGCAGGGTCA
Cas9 WT Control	FWD	AAGGGAGCTGCAGTGGAGTA
	REV	CCGAAAATCTGTGGGAAGTC
CreERT2	FWD	GCGGTCTGGCAGTAAAAACTATC
	REV	GTGAAACAGCATTGCTGTCACTT
CreERT2 WT Control	FWD	CTAGGCCACAGAATTGAAAGATCT
	REV	GTAGGTGGAAATTCTAGCATCATCC
Trp53 R172H	FWD	CCATGGCTTGAGTAAGTCTGCA
	REV	GAAACTTTTCACAAGAACCAGATCA
Trp53 R172H WT Control	FWD	AGGTGTGGCTTCTGGCTTC
	REV	GAAACTTTTCACAAGAACCAGATCA
Trp53 flox	FWD	GGTTAAACCCAGCTTGACCA
	REV	GGAGGCAGAGACAGTTGGAG
Pten flox	FWD	GTCACCAGGATGCTTCTGAC
	REV	GTCACCAGGATGCTTCTGAC
Pten flox WT Control	FWD	GTCACCAGGATGCTTCTGAC
	REV	GTCACCAGGATGCTTCTGAC
**Recombination confirming PCR primer**		
**Target**	**Direction**	**Sequence 5' → 3'**
Trp53 LOH	FWD	AGCCTGCCTAGCTTCCTCAGG
	REV	CAGTGTGGCTATGTCTCCAAG
Trp53 flox	FWD	CACAAAAACAGGTTAAACCCA
	REV	GAAGACAGAAAAGGGGAGGG
Pten flox	FWD	GTCACCAGGATGCTTCTGAC
	REV	TGTGAACTCCCACCAATGAA

Table S1 continued

qPCR primer		
**Gene**	**Direction**	**Sequence 5' → 3'**
Pten	FWD	CCAAGTCCAGAGCCATTTCCA
	REV	TCATTACACCAGTCCGTCCC
Lgals9	FWD	CTGGAATCCCTCCTGTGGTGTA
	REV	CCTCGTAGCATCTGGCAAGACA
Nectin2	FWD	GCCATACTGACCTGTGATGTACG
	REV	TCCACAGAGTGGACAAGCAGCT
Cd274	FWD	TTGCTACGGGCGTTTACTATC
	REV	TCCCGTTCTACAGGGAATCT
Ccl5	FWD	AGCTGCCCTCACCATCATCCTCACT
	REV	CACTTCTTCTCTGGGTTGGCACAC
Ccl2	FWD	AGGTGTCCCAAAGAAGCTGT
	REV	GACCTTAGGGCAGATGCAGTT
Cxcl5	FWD	CGGAGCTGCGTTGTGTTTG
	REV	TTCCGCTTAGCTTTCTTTTTGTCA
Cxcl16	FWD	TGAGGCTGAGGCAAATGAGAAACA
	REV	GAAGACAATGGCCAGGAGGGACAG
Il17rc	FWD	AGAGGAGTTGCTGCAAGACT
	REV	CCCATAGCGATCCCATGTTG
Il33	FWD	TCGGGTACCAAGCATGAAGAGAAC
	REV	TCCACACCGTCGCCTGATTG
Isg15	FWD	ACAGTGATGCTAGTGGTACA
	REV	AAGACCTCATAGATGTTGCT
Tnfα	FWD	ATCTTCTCAAAATTCGAGTG
	REV	ACCACTAGTTGGTTGTCTTT

Supplementary Table S1. PCR primer information.
Genotyping PCR, Recombination confirming PCR, and qPCR primers used in this study.

Table S2

Supplementary Table S2. Mouse cytokine array data.

Please refer to the file of Table S2.
