# Supplementary material for: PTEN loss drives p53 LOH and immune evasion in a novel urothelial organoid model harboring p53 missense mutations
Source: Oncogene. 2025 Feb 22;44(19):1336–49. doi: 10.1038/s41388-025-03311-5 (PMC12052601; doi:10.1038/s41388-025-03311-5)
Supplement: Supplementary file 2 — Supplementary Materials and Methods [file 41388_2025_3311_MOESM2_ESM.pdf]

Supplementary Materials and Methods

Establishment of mouse-derived and tumor-derived organoids

All animal experiments were carried out according to the protocol approved by the Committee on Animal Experimentation of Kyoto University (Approval No.: Med Kyo 21223). *FVB.Cg-Tg (KRT5-cre/ERT2)2Ipc/JeldJ* (*$Krt5^{CreERT2}$*), *129S-$Trp53^{tm2Tyj/J}$* (*$Trp53^{LSL\text{-}R172H}$*), *B6;129-Gt(ROSA)$26Sor^{tm1(CAG\text{-}cas9*,\text{-}EGFP)Fezh}$/J* (*$Rosa26^{LSL\text{-}Cas9\text{-}EGFP}$*), and *B6.129P2-$Trp53^{tm1Brn}$/J* (*$Trp53^{flox}$*) strains were obtained from the Jackson Laboratory (stock numbers 018394, 008652, 024857, and 008462, respectively). The *$Pten^{flox}$* strain was provided by University of Toronto, Toronto, Canada (1). *$Krt5^{CreERT2}$* mice and *$Rosa26^{Cas9\text{-}EGFP}$* mice were backcrossed with the *$Trp53^{R172H}$*, *$Trp53^{flox}$*, *and $Pten^{flox}$* mice. Mice were maintained on a predominantly C57BL/6 background. When treated with tamoxifen, the mated mice expressed the Trp53 R172H mutation or lost Trp53 or Pten expression with Cas9-EGFP expression, specifically under control of the *Krt5* promoter. Tamoxifen (Sigma-Aldrich, St. Louis, MO, USA, T5648) was delivered at a dose of 0.3 mg/g body weight by oral gavage for 3 consecutive days. Seven days after tamoxifen delivery *in vivo*, organoids were generated from the bladder urothelium. The urothelium was microscopically scraped, cut into small pieces, and digested with 5 mg/mL collagenase Type I (Thermo Fisher Scientific, Waltham, MA, USA, 17100-017) in a thermoblock with 400 rpm shaking at 37°C for 1 hour. For tumor-derived organoids, tumor tissue was taken from the mouse, minced into small pieces, and

digested with collagenase in the same manner. The cell suspension was collected, dissociated with TrypLE (Thermo Fisher Scientific, 12605036) with 400 rpm shaking at 37°C for 10 minutes, filtered through a 70-μm strainer, and centrifuged at 500×g for 5 minutes at 4°C. The pellet was washed with Advanced DMEM/F12 medium (Gibco, Thermo Fisher Scientific, 12634010) and the cells were embedded in growth factor-reduced Matrigel (Corning, Corning, NY, USA, 354230). Matrigel-cell suspensions (10,000 cells/50 μL, containing 80% Matrigel and 20% Advanced DMEM/F12 medium per drop) were plated in 24-well plates, allowed to settle in a humidified incubator at 37°C/5% CO_2 for 20 minutes, and overlaid with 500 μL organoid medium. The medium was replaced every 2–3 days and the organoids were split every 7 days. Organoids were digested with TrypLE with 400 rpm shaking at 37°C for 10 minutes, washed with Advanced DMEM/F12, and plated with Matrigel, as stated above. The organoids were frozen in CP-1 (KYOKUTO, Tokyo, Japan) (50% Advanced DMEM/F12 medium and 50% CP-1) and could be recovered efficiently. The organoids were maintained in culture with organoid medium, as described below, following previous studies (2-4): Advanced DMEM/F12 medium with 1% penicillin/streptomycin and 1% HEPES, 2 mM GlutaMAX (Gibco, 35050061), 0.125 ng/μL human epidermal growth factor (EGF) (Peprotech, Cranbury, NJ, USA, AF-100-15), 0.1 ng/μL human Noggin (Peprotech, 120-10C), 0.5 ng/μL human R-spondin-1 (Peprotech, 120-38), 10 mM nicotinamide (Sigma-Aldrich, N0636), 500 nM A83-01 (Tocris Bioscience, Bristol, UK, 2939),

1× B27 (Gibco, 12587010), and 1.25 mM N-acetylcysteine (Sigma-Aldrich, 616-91-1). After an organoid strain was newly established from a mouse, Krt5-lineage cells were isolated by FITC channels to gate the GFP+ population using a BD FACS Aria II instrument (BD Biosciences, Franklin Lakes, NJ, USA) after two passages. When using nutlin-3 (Chemscene, Monmouth Junction, NJ, USA, CS-0296), 5 μM was added after the passage, followed by culturing in nutlin-containing media. When assessing cell proliferation, organoids were digested with TrypLE as described above, then the number of cells was counted with a TC20 Automated Cell Counter (Bio-Rad Laboratories, Hercules, CA, USA).

Adeno-associated virus (AAV) vector cloning and production

The PrecisionX^{TM} Multiplex gRNA Cloning Kit (System Biosciences, Palo Alto, CA, USA) and AAVpro Helper Free System (Takara Bio, Shiga, Japan) were used for AAV cloning and production, respectively. During the cloning process, single-guide (sg) Control or sgTrp53 and sgRNA of targeted gene with tdTOMATO were inserted into the cassettes. The recombinant expression plasmid was co-transfected into AAVpro 293T cells (Takara Bio) with pHelper and pAAV-RC (carrying the AAV2 replication and capsid genes) using the TransIT-293 Transfection Reagent (MIR2704, Mirus Bio, Madison, WI, USA). The AAV collection and purification steps were performed according to the manufacturer’s suggested protocol.

sgRNA sequences: Control: GGCAGAAGGAACACAGGCTC; Trp53 (same for Trp53^{R172H}): GTGTAATAGCTCCTGCATGG; Pten #1: CGCGGCGGGAGGACAAGTTC; Pten #2: ATCAGGGAGTCACAATTCCC; Kmt2c #1: GTTACTCCACTAAAGCGTGC; Kmt2c #2: GTTATGAAATCAGTGCCAAC.

CRISPR/Cas9 gene editing

Purified AAV (10 μL) with the sgRNA of the specific target gene was added into the organoid medium immediately following passaging. After the transfection level was confirmed by examining tdTOMATO expression with a fluorescence microscope, the organoids were sorted by FITC/Gm-PE channels to gate the GFP and tdTOMATO double-positive population. Gene editing was confirmed using Sanger sequencing with polymerase chain reaction (PCR) products of the CRISPR targeted sites.

PCR primer sequences: Trp53 (same for Trp53^{R172H}) Fw: AGGCTTAGAGGTGCAAGCTG; Trp53 (same for Trp53^{R172H}) Rv: TCCCTAAGCCCAAGAGGAA; Pten #1 Fw: TGCCAAAACCAACAAACAAA; Pten #1 Rv: GGGCAGGTGAGTCTGCTTAC; Pten #2 Fw: GCCTTTGCTTATTGGGTTCA; Pten #2 Rv: AGTGCCACGGGTCTGTAATC; Kmt2c #1 Fw: TCCCCCTCCTAGTTCCTTGT; Kmt2c #1 Rv: TGAGCCTCCAAATCTAGGATG; Kmt2c #2 Fw: TGCTGTACCACCTCCTCCTC; Kmt2c #2 Rv: AAGCAAAGCTACCTGGGAAA.

Mouse experiments

Female 6- to 8-week-old BALB/cAJcl-nu/nu mice and C57BL/6NJcl mice (CLEA Japan, Inc., Tokyo, Japan) were purchased for transplantation experiments. Dissociated organoid cells (1×10^4–1×10^6 cells/mouse) were inoculated into the subcutaneous tissue, bladder wall, and renal subcapsular layer of the mice (100 μL per site, containing 50% Matrigel). For orthotopic injections, 100 μL saline was first injected between the urothelial layer and muscle layer, and then cells with Matrigel were injected into the pre-made space. Tumor sizes were measured every week until the tumor reached 7 mm in size, the tumor volume reached 1000 mm^3, or after 13 weeks of follow-up. Tumor formation was defined as the tumor growing over 100 mm^3. The mice were housed in the specific-pathogen-free facility of Kyoto University, Japan, with a 12-hour light/dark cycle at 23°C ± 2°C (room temperature) and relative humidity of 50 ± 20%, and given *ad libitum* access to food and water for the duration of the study.

Trp53 LOH PCR

Genomic DNA was extracted from organoids and tumors using the DNeasy Blood & Tissue Kit (Qiagen, Hilden, Germany). To detect the wild-type (WT) Trp53 (290 bp) and mutant Trp53 (330 bp), DNA was amplified by PCR. Primer information is shown in Supplementary Table S1.

Digital PCR

QuantStudio^{TM} 3D Digital PCR (Thermo Fisher Scientific) was used to quantify WT Trp53 and mutant Trp53^{R172H}. The probe information is included below:

WT Trp53 (VIC): TGAGACGCTGCCCC; Mutant Trp53^{R172H} (FAM): TGAGACACTGCCCC; Forward primer sequence: CATCTACAAGAAGTCACAGCACATG; Reverse primer sequence: GGAGCAGCGCTCATGGT.

Real-time quantitative PCR

Total RNA was extracted from the cultured organoids or harvested tumors using RNeasy Mini kits (Qiagen) according to the manufacturer's protocols. Complementary DNA was synthesized from 1 μg of total RNA using the ReverTra Ace qPCR RT Kit (TOYOBO, Osaka, Japan). Real-time quantitative RT-PCR was performed with SYBR Green PCR Master Mix (Life Technologies, Carlsbad, CA, USA) and the Thermal Cycler Dice Real Time System II (Takara). The thermal cycling conditions were as follows: initial denaturation at 95°C for 10 minutes; 40 cycles of denaturation at 94°C for 15 s, annealing at 58°C for 30 s, and extension at 72°C for 30 s. Relative expression levels were determined by normalization to data for the housekeeping gene *Gapdh*. Each reaction was performed in triplicate. The primers used are listed in Supplementary Table S1. The ΔΔCt method was used to determine the relative expression levels of the genes of interest.

Western blot analysis

Organoids were lysed with RIPA buffer containing proteinase inhibitors. Protein samples were resolved by SDS-PAGE and transferred to polyvinylidene difluoride membranes (Millipore, Bedford, MA, USA) using a Mini Trans-Blot Cell system (Bio-Rad Laboratories). Membranes were blocked with 5% bovine serum albumin (BSA) diluted in Tris-buffered saline, Tween 20 (TBST) at room temperature for 1 hour, then incubated with primary antibodies diluted in 1% BSA/TBST at 4°C overnight. They were then incubated with horseradish peroxidase-conjugated secondary antibodies diluted in 1% BSA/TBST at room temperature for 1 hour and developed for reading by enhanced chemiluminescence (SuperSignal West Pico Chemiluminescent Substrate, Thermo Fisher Scientific). Images were acquired with the LAS-4000 imaging system (Fujifilm Life Science, Tokyo, Japan). Primary antibodies for Pten (9188, Cell Signaling Technology, Danvers, MA, USA, 1:1000), p-Akt (4060, Cell Signaling Technology, 1:2000), t-Akt (9272, Cell Signaling Technology, 1:1000), and Vinculin (13901, Cell Signaling Technology, 1:1000) were used. Anti-rabbit IgG (7074, Cell Signaling Technology, 1:25,000) was used as a secondary antibody.

Immunohistochemistry (IHC)

Paraffin sections were cut (5 mm thick), dewaxed in xylene, and treated with an ethanol gradient. The sections were stained with hematoxylin and eosin (H&E) or with antibodies against cytokeratin 5 (ab52635, 1:100; Abcam, Cambridge, UK), forkhead box A1 (Foxa1) (ab170933, 1:1000; Abcam), p63 (ab124762, 1:500; Abcam), CK20 (ab97511, 1:800; Abcam), Ki67 (ab15580, 1:800; Abcam), p53 (NCL-p53-CM5p, 1:500; Leica Biosystems, Buffalo Grove, IL, USA), CD8 (ab209775, 1:2000; Abcam), CD206 (ab64693, 1:100,000; Abcam), and Foxp3 (ab215206, 1:500; Abcam).

Whole exome sequencing (WES) analysis

This study was approved by the institutional Review Board at Kyoto University Graduate School of Medicine (#G0052-17). DNA samples were purified from human bladder tumor tissues and blood samples (n=10), as well as from mouse bladder tumor and healthy liver tissues (n=6). Paired-end sequencing for both human and mouse samples was then performed using the Illumina (San Diego, CA, USA) HiSeq 2000 platform. Exonic regions were captured using the Agilent SureSelectXT Human All Exon for human and SureSelectXT Mouse All Exon V1 for mouse according to the manufacturer’s instructions (Agilent, Santa Clara, CA, USA). The obtained sequence data were processed using cutadapt (version 1.1) (5) and trimmomatic (version 0.32) (6) to remove adapters and filter out low-quality reads. The filtered reads were then mapped to GRCh37 and GRCm37 using the Burrows-Wheeler

Alignment tool (version 0.7.10) (7). Preprocessing was performed using the Genome Analysis Toolkit (version 2.3.0) (8) and Picard tools (version 1.115) (9). Variant calling was performed using Mutect (version 1.1.7) (10) on paired normal and tumor data. Detected variants were annotated for their impact using SnpEFF (11).

RNA-sequencing (RNA-Seq) analysis

Total RNA was extracted from organoids as described above. Paired-end transcriptome sequencing was then performed using the NovaSeq 6000 platform (Illumina). For the raw read data, adapter removal and filtering of low-quality reads were performed using trimmomatic (version 0.39). The processed reads were then mapped to mm10 using STAR (version 2.7.8) (12). Gene expression quantification was performed with RSEM (version 1.3.3) (13) and gene-level count data were generated using the summarizeToGene function from tximport (version 1.22) (14). Using the GENCODE annotation data (gencode.vM25.annotation.only.protein_coding.tsv), genes with a gene_type of "protein coding" were selected.

Before conducting Gene Set Enrichment Analysis (GSEA), the count data were processed using edgeR (version 3.36) (15). Lowly expressed genes were filtered out with the filterByExpr library in edgeR, followed by normalization using the trimmed mean of M-values method and conversion to $\log_2$CPM values.

GSEA was conducted using the gseapy library (version 0.10.8) (16) in Python. The hallmark gene sets were sourced from The Molecular Signatures Database (17). The Cancer Genome Atlas (TCGA) 2017 cluster gene sets were obtained from Supplementary Table S3 of a previous study (18). We used the biomaRt package (19) for conversion to mouse gene symbols.

TCGA data analysis

Somatic variant data were obtained from the TCGA_BLCA project on the National Cancer Institute Genomic Data Commons (GDC) portal (20), specifically the MAF files generated by the Mutect2 variant aggregation and masking workflow. Copy number data were sourced from cBioportal (21) by selecting the "TCGA, Cell 2017" dataset and filtering for the Tp53 gene. "Gene-Level Copy Number Scores data" were then downloaded (as of September 2020). Clinical information for each TCGA case, such as event (the number of days to death or to the last follow-up), tumor stage, and pathological stage, were obtained from the TCGA_BLCA project on the GDC data portal. Subtype information was obtained from a supplemental table (S2.19) in a previous report (22).

For case classification, the somatic mutation data were initially used. If the variant_type was either *Frame_Shift_Del*, *Frame_Shift_Ins*, *Nonsense_Mutation*, *Splice_Region*, or *Splice_Site*, then the allele was defined as a deletion (NSFS). If the

variant_type was *In_Frame_Del*, *Nonstop_Mutation*, or *Missense_Mutation*, then the allele was defined as a mutation (MS). If the variant_type was *3'UTR*, *5'UTR*, *Intron*, *Silent*, or the same as the reference, then the allele was defined as WT. Furthermore, following the methods of a prior study (23), if the proportion of mutant reads to depth was 0.65 or more, then it was considered copy-neutral LOH (HOM). If it was less than 0.65, then it was designated as no LOH (HET). For copy numbers, on the basis of the Copy Number Score, a score of -2 was defined as DEL (homozygous deletion), -1 as C1 (heterozygous deletion), and 0 as C2 (diploid).

Copy number analysis

Genomic DNA was extracted from organoids, as described above. Paired-end Whole genome sequencing (WGS) was performed using the NovaSeq 6000 platform. Then, copy number variation analysis was performed using CNV kit Ver 0.9.9 (24).

Seahorse flux analyses

The oxygen consumption rate (OCR) and extracellular acidification rate (ECAR) of the organoids were measured using the Seahorse XFe96 Extracellular Flux Analyzer (Agilent Technologies) according to a previous report (25). First, the number of cells per well was optimized to keep the baseline OCR above 100 pmol/min. Then, organoids with different

genotypes were plated in 96-well Seahorse plates 3 days before the measurement. Organoids were seeded with optimal cell numbers in 3 μL drops (40% Matrigel) with six wells per genotype. The OCR and ECAR values were measured at baseline and after injections of oligomycin (1 μM) and Rotenone/Antimycin (2 μM). All OCR and ECAR values were normalized to the DNA amount using the DNA Micro kit (Qiagen).

Mouse cytokine array for organoids

Tumor-derived organoids were plated with 10,000 cells/drop (50% Matrigel) in 24-well plates with 500 μL organoid medium. After 5 days, the medium was replaced with 500 μL of organoid medium without Noggin and R-spondin. Another 24 hours later, the supernatants were collected and centrifuged at 400 x g at 4°C for 10 minutes. The supernatants were applied to Quantibody Mouse Cytokine Array Q1000 slides (RayBiotech, Norcross, GA, USA) and sent to RayBiotech for cytokine profiling.

Statistical analyses

Statistical analyses were performed using Wilcoxon's nonparametric analysis. The log-rank test was used for tumor formation rate analyses (Figs. S5C, 4A/B/D). Multiple regression analysis was used to analyze the Seahorse ATP assay data (Fig. 3C). *P*-values < 0.05 were considered statistically significant. R 4.2.2, GraphPad Prism9, and JMP Pro 15 were used for

statistical analysis and data visualization. Data are expressed as the mean ± standard deviation, unless otherwise indicated.

References

1. Suzuki A, Yamaguchi MT, Ohteki T, Sasaki T, Kaisho T, Kimura Y, et al. T cell-specific loss of Pten leads to defects in central and peripheral tolerance. Immunity. 2001;14:523-34.
2. Halstead AM, Kapadia CD, Bolzenius J, Chu CE, Schriefer A, Wartman LD, et al. Bladder-cancer-associated mutations in. Elife. 2017;6.
3. Mullenders J, de Jongh E, Brousali A, Roosen M, Blom JPA, Begthel H, et al. Mouse and human urothelial cancer organoids: A tool for bladder cancer research. Proc Natl Acad Sci U S A. 2019;116:4567-74.
4. Santos CP, Lapi E, Martínez de Villarreal J, Álvaro-Espinosa L, Fernández-Barral A, Barbáchano A, et al. Urothelial organoids originating from Cd49f. Nat Commun. 2019;10:4407.
5. Martin M. Cutadapt removes adapter sequences from high-throughput sequencing reads. *EMBnet.journal*. 2011;17:10-12.
6. Bolger AM, Lohse M, Usadel B. Trimmomatic: a flexible trimmer for Illumina sequence data. Bioinformatics. 2014;30:2114-20.
7. Li H, Durbin R. Fast and accurate short read alignment with Burrows-Wheeler transform. Bioinformatics. 2009;25:1754-60.
8. Van der Auwera GA, O'Connor BD. *Genomics in the Cloud: Using Docker, GATK, and WDL in Terra*. "O'Reilly Media, Inc."; 2020.
9. Picard. Picard. Accessed November 1, 2023. http://broadinstitute.github.io/picard.
10. Cibulskis K, Lawrence MS, Carter SL, Sivachenko A, Jaffe D, Sougnez C, et al. Sensitive detection of somatic point mutations in impure and heterogeneous cancer samples. Nat Biotechnol. 2013;31:213-9.
11. Cingolani P, Platts A, Wang lL, Coon M, Nguyen T, Wang L, et al. A program for annotating and predicting the effects of single nucleotide polymorphisms, SnpEff: SNPs in the genome of Drosophila melanogaster strain w1118; iso-2; iso-3. Fly (Austin). 2012;6:80-92.
12. Dobin A, Davis CA, Schlesinger F, Drenkow J, Zaleski C, Jha S, et al. STAR: ultrafast universal RNA-seq aligner. Bioinformatics. 2013;29:15-21.
13. Li B, Dewey CN. RSEM: accurate transcript quantification from RNA-Seq data with or without a reference genome. BMC Bioinformatics. 2011;12:323.
14. Soneson C, Love MI, Robinson MD. Differential analyses for RNA-seq: transcript-level estimates improve gene-level inferences. F1000Res. 2015;4:1521.
15. Robinson MD, McCarthy DJ, Smyth GK. edgeR: a Bioconductor package for differential expression analysis of digital gene expression data. Bioinformatics. 2010;26:139-40.
16. Fang Z, Liu X, Peltz G. GSEApy: a comprehensive package for performing gene set enrichment analysis in Python. Bioinformatics. 2023;39:btac757.
17. Subramanian A, Tamayo P, Mootha VK, Mukherjee S, Ebert BL, Gillette MA, et al. Gene set enrichment analysis: a knowledge-based approach for interpreting genome-wide expression profiles. Proc Natl Acad Sci U S A. 2005;102:15545-50.

18. Masuda N, Murakami K, Kita Y, Hamada A, Kamada M, Teramoto Y, et al. Trp53 Mutation in Keratin 5 (Krt5)-Expressing Basal Cells Facilitates the Development of Basal Squamous-Like Invasive Bladder Cancer in the Chemical Carcinogenesis of Mouse Bladder. Am J Pathol. 2020;190:1752-62.
19. Durinck S, Spellman PT, Birney E, Huber W. Mapping identifiers for the integration of genomic datasets with the R/Bioconductor package biomaRt. Nat Protoc. 2009;4:1184-91.
20. Grossman RL, Heath AP, Ferretti V, Varmus HE, Lowy DR, Kibbe WA, et al. Toward a Shared Vision for Cancer Genomic Data. N Engl J Med. 2016;375:1109-12.
21. Cerami E, Gao J, Dogrusoz U, Gross BE, Sumer SO, Aksoy BA, et al. The cBio cancer genomics portal: an open platform for exploring multidimensional cancer genomics data. Cancer Discov. 2012;2:401-4.
22. Robertson AG, Kim J, Al-Ahmadie H, Bellmunt J, Guo G, Cherniack AD, et al. Comprehensive Molecular Characterization of Muscle-Invasive Bladder Cancer. Cell. 2017;171:540-56.e25.
23. Parikh N, Hilsenbeck S, Creighton CJ, Dayaram T, Shuck R, Shinbrot E, et al. Effects of TP53 mutational status on gene expression patterns across 10 human cancer types. J Pathol. 2014;232:522-33.
24. Talevich E, Shain AH, Botton T, Bastian BC. CNVkit: Genome-Wide Copy Number Detection and Visualization from Targeted DNA Sequencing. PLoS Comput Biol. 2016;12:e1004873.
25. Ludikhuize MC, Meerlo M, Burgering BMT, Rodríguez Colman MJ. Protocol to profile the bioenergetics of organoids using Seahorse. STAR Protoc. 2021;2:100386.
